# Enhancing Late‐Life Survival and Mobility via *Mitohormesis* by Reducing Mitochondrial Calcium Levels

**DOI:** 10.1111/acel.70247

**Published:** 2025-09-26

**Authors:** Doruntina Bresilla, Ines Tawfik, Martin Hirtl, Sonja Gabrijelčič, Julian Ostaku, Fabienne Mossegger, Lia Wurzer, Susanne Lederer, Katarina Kalinova, Ernst Malle, Markus Schosserer, Kim Zarse, Michael Ristow, Corina T. Madreiter‐Sokolowski

**Affiliations:** ^1^ Division of Molecular Biology and Biochemistry Medical University of Graz Graz Austria; ^2^ Center for Pathobiochemistry and Genetics Medical University of Vienna Vienna Austria; ^3^ Charité—Universitätsmedizin Berlin, Corporate Member of Freie Universität Berlin and Humboldt Universität Zu Berlin Institute for Experimental Endocrinology Berlin Germany; ^4^ BioTechMed‐Graz Graz Austria

**Keywords:** aging, *C. elegans*, lifespan, longevity, mitochondria, reactive oxygen species

## Abstract

Mitochondrial calcium (Ca^2+^) homeostasis plays a critical role in aging and cellular fitness. In the search for novel antiaging approaches, we explored how genetic and pharmacological inhibition of mitochondrial Ca^2+^ uptake influences the lifespan and health of *Caenorhabditis elegans* . Using live‐cell imaging, we demonstrate that RNA interference‐mediated knockdown of *mcu‐1*, the nematode ortholog of the mitochondrial Ca^2+^ uniporter (MCU), reduces mitochondrial Ca^2+^ levels, thereby extending lifespan and preserving motility during aging, while compromising early‐life survival. This longevity benefit requires intervention before day 14 and coincides with a transient increase in reactive oxygen species (ROS), which activates pathways involving *pmk‐1*, *daf‐16*, and *skn‐1*, orthologs of human p38 mitogen‐activated protein kinase (p38 MAPK), forkhead box O (FOXO), and nuclear factor erythroid 2–related factor 2 (NRF2), respectively. This pathway promotes antioxidant defense mechanisms and preserves mitochondrial structure and function during aging, maintaining larger, more interconnected mitochondria and restoring the oxidized/reduced nicotinamide adenine dinucleotide (NAD^+^/NADH) ratio and oxygen consumption rates to youthful levels. Pharmacological inhibition of mitochondrial Ca^2+^ uptake using the MCU inhibitor mitoxantrone mirrors the effects of *mcu‐1* knockdown, extending lifespan and improving fitness in aged nematodes. In human foreskin fibroblasts, short‐term mitoxantrone treatment also transiently elevates ROS production and induces enhanced expression and activity of antioxidant defense enzymes, underscoring the translational relevance of findings from nematodes to human cells. Our findings suggest that modulation of mitochondrial Ca^2+^ uptake induces *mitohormesis* through ROS‐mediated signaling, promoting improved longevity and healthspan in nematodes, with possible implications for healthy aging in humans.

## Graphical abstract

RNAi knockdown (KD) of *mcu‐1* or mitoxantrone treatment lowers mitochondrial Ca^2+^, extends lifespan, and preserves motility during aging. Benefits require early intervention and involve a transient ROS increase that activates a *pmk‐1*, *daf‐16*, and *skn‐1* pathway, enhancing stress defense and mitochondrial integrity while reducing early‐life survival.

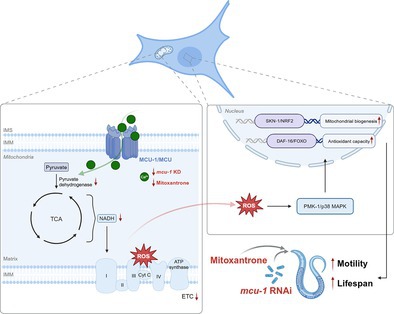

## 1 Introduction

Mitochondrial calcium (Ca^2+^) plays a central role in regulating numerous cellular functions, including metabolism, signaling, and the balance between cellular survival and death. Thereby, mitochondrial Ca^2+^ homeostasis is a delicate balance. Both excessive and insufficient Ca^2+^ uptake can disrupt mitochondrial function and may lead to cellular dysfunction. For instance, mitochondrial Ca^2+^ is essential to boost the activity of dehydrogenases of the tricarboxylic acid (TCA) cycle (Tawfik et al. 2022), while excessive mitochondrial Ca^2+^ load leads to apoptosis (Madreiter‐Sokolowski et al. 2016).

As a result, dysregulation of mitochondrial Ca^2+^ can significantly accelerate physiological decline and is increasingly recognized as a major contributor to aging and age‐related diseases, including neurodegeneration, cardiovascular disease, and metabolic disorders (Madreiter‐Sokolowski et al. 2024). During the aging process, changes in the expression and function of proteins that regulate mitochondrial Ca^2+^ handling (Madreiter‐Sokolowski et al. 2019; Uzhachenko et al. 2017; Yang et al. 2021) lead to an imbalance in mitochondrial Ca^2+^ homeostasis, resulting in age‐related deterioration. For example, a decline in mitochondrial Ca^2+^ retention capacity has been linked to age‐related impairments in muscle function (Cefis et al. 2025). In addition, elevated basal mitochondrial Ca^2+^ levels—potentially due to increased physical interaction between the endoplasmic reticulum and mitochondria—have been associated with mitochondrial dysfunction and are increasingly recognized as both features and potential drivers of cellular senescence (Madreiter‐Sokolowski et al. 2019; Margand et al. 2025). Interestingly, also in *Caenorhabditis elegans (C. elegans)*, increased mitochondrial Ca^2+^ levels have been shown to contribute to health decline, pointing toward a detrimental role of increased mitochondrial Ca^2+^ levels in aged organisms (Higashitani et al. 2023).

Aging‐related alterations in mitochondrial Ca^2+^ handling not only affect different organisms but also various cell types, contributing to age‐related organ dysfunction. For example, mitochondrial Ca^2+^ overload in aged neurons is associated with glutamate‐induced excitotoxicity and excessive reactive oxygen species (ROS) generation—events that ultimately trigger apoptotic neuronal death and contribute to diseases such as Alzheimer's and Parkinson's disease (Madreiter‐Sokolowski et al. 2024). Mitochondrial Ca^2+^ dysregulation is also a key factor in ischemia–reperfusion events, where mitochondrial Ca^2+^ overload leads to the opening of the mitochondrial permeability transition pore, triggering cell death pathways in cardiomyocytes and cardiac injury after reperfusion (Rozich et al. 2025). Moreover, pancreatic beta‐cell insulin secretion relies on mitochondrial Ca^2+^ uptake, which links glucose metabolism to ATP production and triggers Ca^2+^‐dependent exocytosis of insulin, a process that is strongly affected by age‐related alterations in mitochondrial Ca^2+^ homeostasis, potentially serving as a trigger for the development of metabolic diseases like type 2 diabetes mellitus (Mammucari et al. 2018).

Therefore, a tight control of mitochondrial Ca^2+^ uptake and homeostasis is essential to maintain intact cellular function and prevent age‐related pathologies. This regulation is primarily mediated by a specialized set of proteins that govern mitochondrial Ca^2+^ entry. The outer mitochondrial membrane is largely permeable to Ca^2+^ through the voltage‐dependent anion channel (Shoshan‐Barmatz et al. 2018). In contrast, the uptake of Ca^2+^ into the mitochondrial matrix is tightly regulated by the mitochondrial calcium uniporter (MCU), a highly selective channel located in the inner mitochondrial membrane (Kirichok et al. 2004). The MCU is functionally linked to the essential MCU regulator (EMRE) (Sancak et al. 2013), which connects it to the regulatory proteins mitochondrial Ca^2+^ uptake 1 and 2 (MICU1 and MICU2) (Perocchi et al. 2010; Plovanich et al. 2013). Furthermore, the dominant‐negative pore‐forming subunit MCUb and the scaffold factor MCU regulator 1 modulate mitochondrial Ca^2+^ uptake via MCU (Madreiter‐Sokolowski et al. 2020). Notably, MCU is also a pharmacologically accessible target, and several MCU inhibitors, including the ruthenium red derivatives Ru265 and mitoxantrone, are available. Mitoxantrone has been approved by the Food and Drug Administration (FDA) and the European Medicines Agency for use in humans as an anticancer therapeutic due to its action on DNA topoisomerase II. Additionally, there are reports of the neuroprotective potential of this compound, which could also explain its potential repurposing as a drug to reduce the relapse rate and progression of multiple sclerosis (Marmolejo‐Garza et al. 2023).

In *C. elegans* , MCU‐1 is essential for rapid mitochondrial Ca^2+^ uptake and for the generation of ROS following wounding (Xu and Chisholm 2014). Furthermore, carbachol‐induced muscle stimulation was reported to cause a smaller and shorter mitochondrial Ca^2+^ increase in worms defective in *mcu‐1* (Alvarez‐Illera et al. 2020). Additional known components of the MCU complex in *C. elegans* are *emre‐1* and *micu‐1* (Alvarez‐Illera et al. 2020). While their function remains elusive, the *micu‐1(null)* mutation has been associated with longevity (Jackson et al. 2022).

Mitochondrial Ca^2+^ enhances the activity of dehydrogenases in the TCA cycle (Denton 2009) and increases the production of the reducing equivalents nicotinamide adenine dinucleotide (NADH) and flavin adenine dinucleotide, which fuel the electron transport chain (ETC). While this process is essential for adenosine triphosphate (ATP) production, the transfer of electrons through the ETC also generates ROS as byproducts. ROS, such as superoxide and hydrogen peroxide (H_2_O_2_), are reactive molecules that can, if not adequately regulated, damage proteins, lipids, DNA, and other biomolecules. Therefore, ROS must be detoxified by mitochondrial superoxide dismutase 2 (SOD2) and cytosolic SOD1, which convert it to H_2_O_2_. This H_2_O_2_ is then further processed to water and oxygen by catalase (CAT) or to water and oxidized glutathione (GSSG) by glutathione peroxidase. Moreover, reduced peroxiredoxins have also been reported to catalyze the reduction of H_2_O_2_ to H_2_O (Madreiter‐Sokolowski et al. 2020). Orthologs for all these human enzymes have also been identified in *C. elegans*, and their role has been extensively investigated regarding life‐ and healthspan‐related signaling cascades in nematodes (Lin et al. 2023). In *C. elegans* , reduced glucose availability has been shown, for instance, to trigger a temporary increase in ROS, which in turn enhances the organism's resistance to oxidative stress and extends lifespan through mitochondrial adaptation—a phenomenon called *mitohormesis* (Schulz et al. 2007) and not restricted to nematodes. In mice, time‐restricted depletion of SOD2 during embryonic development associated with significant oxidative stress led to increased mitochondrial biogenesis and expression of antioxidant genes, ultimately resulting in lower ROS levels in the adapted adult animals (Cox et al. 2018). Even in humans, exercise‐induced oxidative stress has been found to improve insulin sensitivity and stimulate the body's own antioxidant defense potential—effects that are blocked when external antioxidants are administered (Ristow et al. 2009). Collectively, these findings highlight that ROS are not merely harmful byproducts of metabolism but also serve as important signaling molecules in different organisms. They activate specific signaling pathways and transcription factors that upregulate genes involved in antioxidant protection. This adaptive response helps against the excessive ROS accumulation associated with aging, which is often driven by mitochondrial dysfunction, such as defects in the ETC (Ristow and Zarse 2010).

Given the central role of mitochondrial Ca^2+^ in regulating the activity of mitochondria, specifically mitochondrial respiration, we assumed that disturbances in mitochondrial Ca^2+^ homeostasis may critically influence redox balance and cellular aging in further consequence. Therefore, this study aimed to investigate how targeted inhibition of mitochondrial Ca^2+^ uptake affects ROS homeostasis and functional decline associated with aging.

## 2 Results

### 2.1 Decreased Mitochondrial Ca^2+^ Levels Prolong Lifespan and Enhance Motility

**FIGURE 1:**
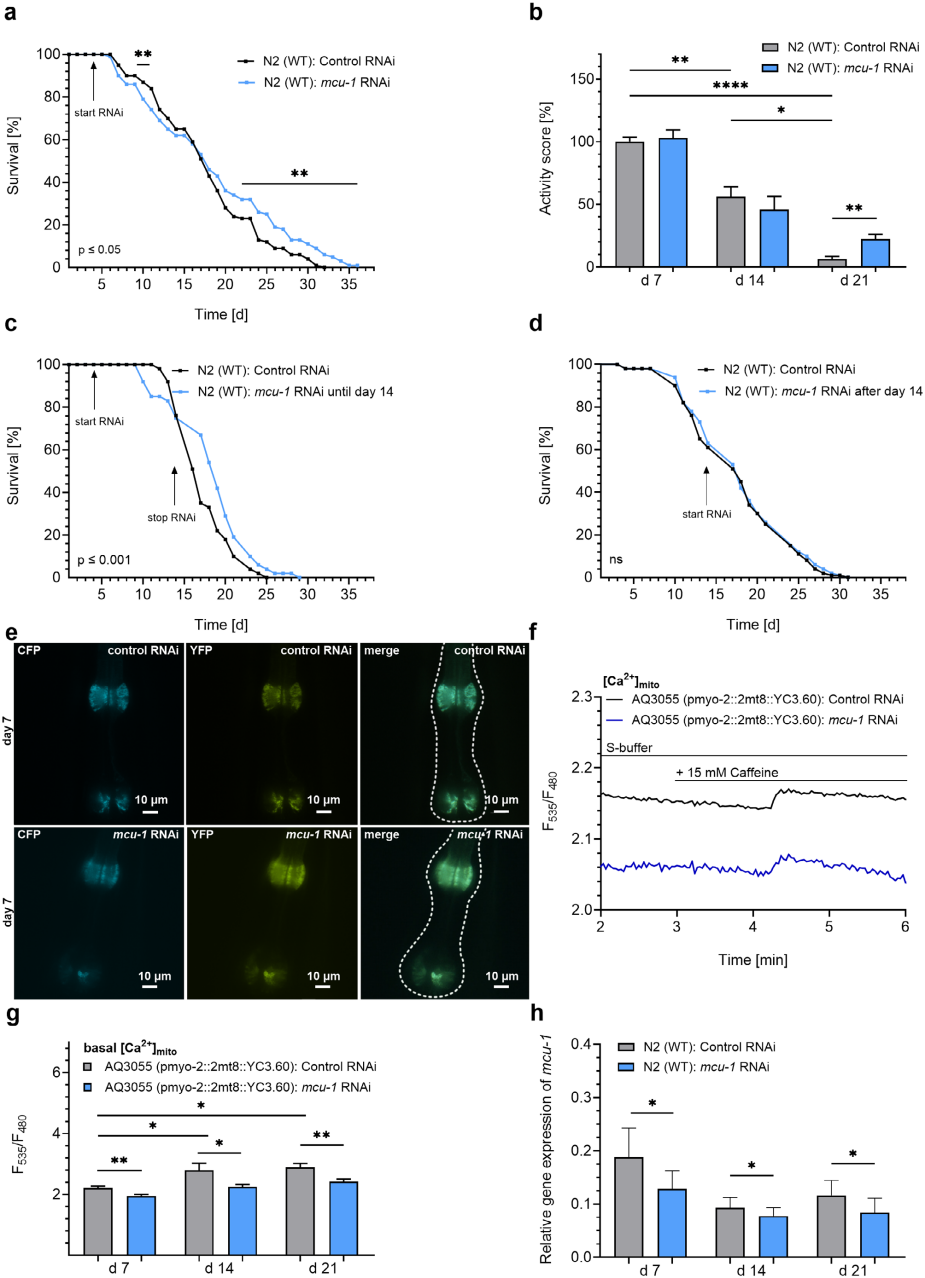
Knockdown of *mcu‐1* prolongs lifespan and motility of *C. elegans* . (a) Lifespan analyses of N2 nematodes in the presence of control RNAi (L4440) (black) and *mcu‐1* RNAi (blue) (*n* = 5). (b) Bar graphs (mean ± SEM) represent activity score (%), based on body bends per min, in N2 nematodes on days 7, 14, and 21 following the treatment with control RNAi (L4440) (gray) or *mcu‐1* RNAi (blue) (*n* = 3). (c) Lifespan analysis of N2 *C. elegans* strain treated with control RNAi (black) or *mcu‐1* RNAi (blue) until day 14. (d) Lifespan analysis of N2 *C. elegans* strain treated with control RNAi (black) or *mcu‐1* RNAi (blue) after day 14. RNAi treatments are indicated by arrows labeled “start RNAi” and, when applicable, “stop RNAi” to mark the beginning and end of the treatment period. (e) Confocal images showing fluorescence emission at 480 nm (CFP) (left) and 535 nm (YFP) (middle), as well as the merge (right), in the pharynx (marked with dashed lines in the merge) of the *C. elegans* strain AQ3055, which expresses the mitochondrial Ca^2+^ sensor YC3.60 under a muscle‐specific promoter, following treatment with either control RNAi (top panels) or RNAi targeting *mcu‐1* (bottom panels) on day 7. (f) Representative curves showing [Ca^2+^]_mito_ levels in the AQ3055 *C. elegans* strain, treated with control RNAi or RNAi against *mcu‐1*, expressing YC3.60 under basal conditions and after stimulation with caffeine [15 mM]. (g) Bar graphs (mean ± SEM) represent basal [Ca^2+^]_mito_ levels of the AQ3055 *C. elegans* strain on days 7, 14, and 21, treated with control RNAi (L4440) (gray) and or *mcu‐1* RNAi (blue) (*n* = 5). (h) Bar graphs (mean ± SEM) represent the relative expression levels of the *mcu‐1* mRNA in N2 nematodes on days 7, 14, and 21, treated with control RNAi (L4440) (gray) and or *mcu‐1* RNAi (blue) (*n* = 9). N‐numbers are presented as biological replicates. For details, see Table 1. For comparing significant distributions between different groups in the lifespan assays, statistical calculations were carried out using the log‐rank test. If applicable, significant differences were assessed via one‐way ANOVA or paired *t*‐test and presented as specific *p* values (*p ≤ 0.05, **p ≤ 0.01, ****p ≤ 0.0001).

To evaluate the broader effects of mitochondrial Ca^2+^ modulation, RNA interference (RNAi)‐mediated knockdown of *mcu‐1* was initiated in N2 nematodes on day 4 of adulthood. While *mcu‐1* RNAi reduced survival in early adulthood, the remaining worms experienced a significant extension in lifespan compared to controls (Figure 1a; for statistical details, see Table 1). Next, we assessed motility, measured as body bends per minute, as an indicator of health. While motility declined significantly with age, N2 nematodes treated with *mcu‐1* RNAi exhibited a marked improvement in late adulthood (day 21) (Figure 1b). To pinpoint when mitochondrial Ca^2+^ modulation is most beneficial, *mcu‐1* knockdown was induced during specific life stages. RNAi treatment initiated before day 14 significantly extended lifespan (Figure 1c, Table 1), while lifespan extension was not observed when *mcu‐1* RNAi was applied after day 14 (Figure 1d, Table 1). These results suggest that early adulthood is a critical period for gaining the beneficial effects of reduced mitochondrial Ca^2+^ uptake. To verify reduced mitochondrial Ca^2+^ levels upon *mcu‐1* knockdown, we performed live‐cell imaging in the nematode strain AQ3055 treated with control RNAi or RNAi against *mcu‐1* on days 7, 14, and 21. This strain expresses the mitochondrial‐targeted Ca^2+^ biosensor YC3.60—a genetically encoded Förster resonance energy transfer (FRET)‐based sensor composed of cyan fluorescent protein (CFP) and yellow fluorescent protein (YFP)—in muscle tissues of the pharynx (Figure 1e, Figure S1). We determined basal mitochondrial Ca^2+^ levels and mitochondrial Ca^2+^ uptake after stimulation with caffeine [15 mM] in worms treated with control or *mcu‐1* RNAi (Figure 1f). Live‐cell imaging revealed a statistical increase in basal mitochondrial Ca^2+^ levels from day 7 to days 14 and 21. In addition, there was a significant reduction in basal mitochondrial Ca^2+^ levels at days 7, 14, and 21 through *mcu‐1* knockdown (Figure 1g). Additionally, caffeine‐induced mitochondrial Ca^2+^ uptake tended to be decreased in nematodes treated with *mcu‐1* RNAi (Figure S1). Quantitative real‐time PCR (qRT‐PCR) confirmed that RNAi‐induced *mcu‐1* knockdown resulted in a significant reduction of mRNA expression levels at days 7, 14, and 21 (Figure 1h).

**TABLE 1:** Lifespan results and statistical analyses.

Strain, RNAi, compound	Maximum lifespan [d + SEM]	Mean lifespan [d + SEM]	*p* values: treatment vs. control	Number of experiments (*n*)	Number of total (and censored) nematodes (*n*)	Change of maximum lifespan [%]
N2: wild type, control RNAi	31.50 ± 0.56	15.9 ± 0.31		4	348 (22)	
N2: wild type, *mcu‐1* RNAi	35.67 ± 0.67	17.8 ± 0.41	0.032	4	379 (42)	13.24
N2: wild type, control RNAi, NAC [100 nM]	27.78 ± 0.74	16.7 ± 0.25		3	402 (26)	
N2: wild type, *mcu‐1* RNAi, NAC [100 nM]	27.44 ± 0.75	16.4 ± 0.24	0.082	3	404 (12)	−1.22
N2: wild type, control RNAi, MitoTEMPO [100 nM]	26.75 ± 0.41	15.6 ± 0.22		3	391 (26)	
N2: wild type, *mcu‐1* RNAi, MitoTEMPO [100 nM]	27.00 ± 0.42	15.4 ± 0.22	0.724	3	391 (29)	0.93
N2: wild type, control RNAi, respective control to treatment from day 14	29.78 ± 0.98	16.4 ± 0.30		3	388 (24)	
N2: wild type, *mcu‐1* RNAi, treatment from day 14	29.67 ± 1.14	16.3 ± 0.32	0.323	3	394 (52)	−0.37
N2: wild type, control RNAi, respective control to treatment until day 14	26.33 ± 0.33	16.6 ± 0.17		4	400 (10)	
N2: wild type, *mcu‐1* RNAi, treatment until day 14	28.92 ± 0.36	15.6 ± 0.21	0.000	4	358 (8)	8.06
KU25: *pmk‐1*(km25), control RNAi	31 ± 1.51	14.9 ± 0.33		3	389 (60)	
KU25: *pmk‐1*(km25), *mcu‐1* RNAi	30.5 ± 1.6	16.0 ± 0.35	0.001	3	408 (62)	−1.61
CF1058: *daf‐16*(mu86), control RNAi	23.50 ± 1.12	10.4 ± 0.22		4	310 (28)	
CF1058: *daf‐16*(mu86), *mcu‐1* RNAi	24.17 ± 0.98	10.3 ± 0.25	0.077	4	296 (27)	2.65
EU31: *skn‐1*(zu135), control RNAi	23.89 ± 1.14	6.65 ± 0.34		3	143 (0)	
EU31: *skn‐1*(zu135), *mcu‐1* RNAi	23.78 ± 1.10	6.80 ± 0.36	0.620	3	140 (0)	−0.46
GA184: *sod‐2*(gk257), control RNAi	31.63 ± 1.0	10.5 ± 0.37		3	243 (17)	
GA184: *sod‐2*(gk257), *mcu‐1* RNAi	31.38 ± 0.84	10.1 ± 0.39	0.527	3	253 (29)	−0.79
WM118: *neIs9*[myo‐3::HA::RDE‐1 + rol‐6(su1006)], control RNAi	28.33 ± 0.59	17.8 ± 0.27		4	451 (45)	
WM118: *neIs9*[myo‐3::HA::RDE‐1 + rol‐6(su1006)], *mcu‐1* RNAi	30.75 ± 0.39	17.4 ± 0.29	0.000	4	450 (47)	8.54
N2: wild type, control RNAi, DMSO [0.001%]	29.5 ± 0.92	19.5 ± 0.28		4	318 (9)	
N2: wild type, control RNAi, mitoxantrone [10 nM]	31.42 ± 1.27	20.4 ± 0.34	0.014	4	320 (36)	6.50
N2: wild type, control RNAi, respective control to mitoxantrone [10 nM] after day 14	29.89 ± 0.56	20.4 ± 0.20		3	320 (19)	
N2: wild type, control RNAi, mitoxantrone [10 nM] after day 14	29.56 ± 0.47	20.2 ± 0.20	0.373	3	320 (6)	−1.10
N2: wild type, control RNAi, respective control to mitoxantrone [10 nM] until day 14	26.11 ± 0.26	16.7 ± 0.17		3	397 (10)	
N2: wild type, control RNAi, mitoxantrone [10 nM] until day 14	29.22 ± 0.46	17.7 ± 0.23	0.000	3	399 (13)	8.41
VP303: *kbIs7*[nhx‐2p::rde‐1 + rol‐6(su1006)], control RNAi	28.33 ± 0.69	18.6 ± 0.22		3	474 (50)	
VP303: *kbIs7*[nhx‐2p::rde‐1 + rol‐6(su1006)], *mcu‐1* RNAi	28.89 ± 0.70	17.1 ± 0.27	0.240	3	453 (55)	1.98
Mir248: *aak‐2*(ok524), control RNAi	19.44 ± 0.29	11.4 ± 0.16		1	326 (28)	
Mir248: *aak‐2*(ok524), *mcu‐1* RNAi	22.33 ± 0.60	13.9 ± 0.19	0.736	1	128 (0)	14.87
Mir14: *sir‐2.1*(ok434), control RNAi	23.40 ± 1.07	11.8 ± 0.23		4	134 (0)	
Mir14: *sir‐2.1*(ok434), *mcu‐1* RNAi	29.00 ± 1.61	14.3 ± 0.35	0.054	4	253 (4)	23.93

In summary, these findings suggest that reducing mitochondrial Ca^2+^ levels through RNAi‐mediated *mcu‐1* knockdown extends lifespan and enhances motility in old nematodes, though it may impair survival during early adulthood.

### 2.2 Reduced Mitochondrial Ca^2+^ Levels Impair TCA Cycle and ETC Activity, Leading to a Transient ROS Rise

**FIGURE 2:**
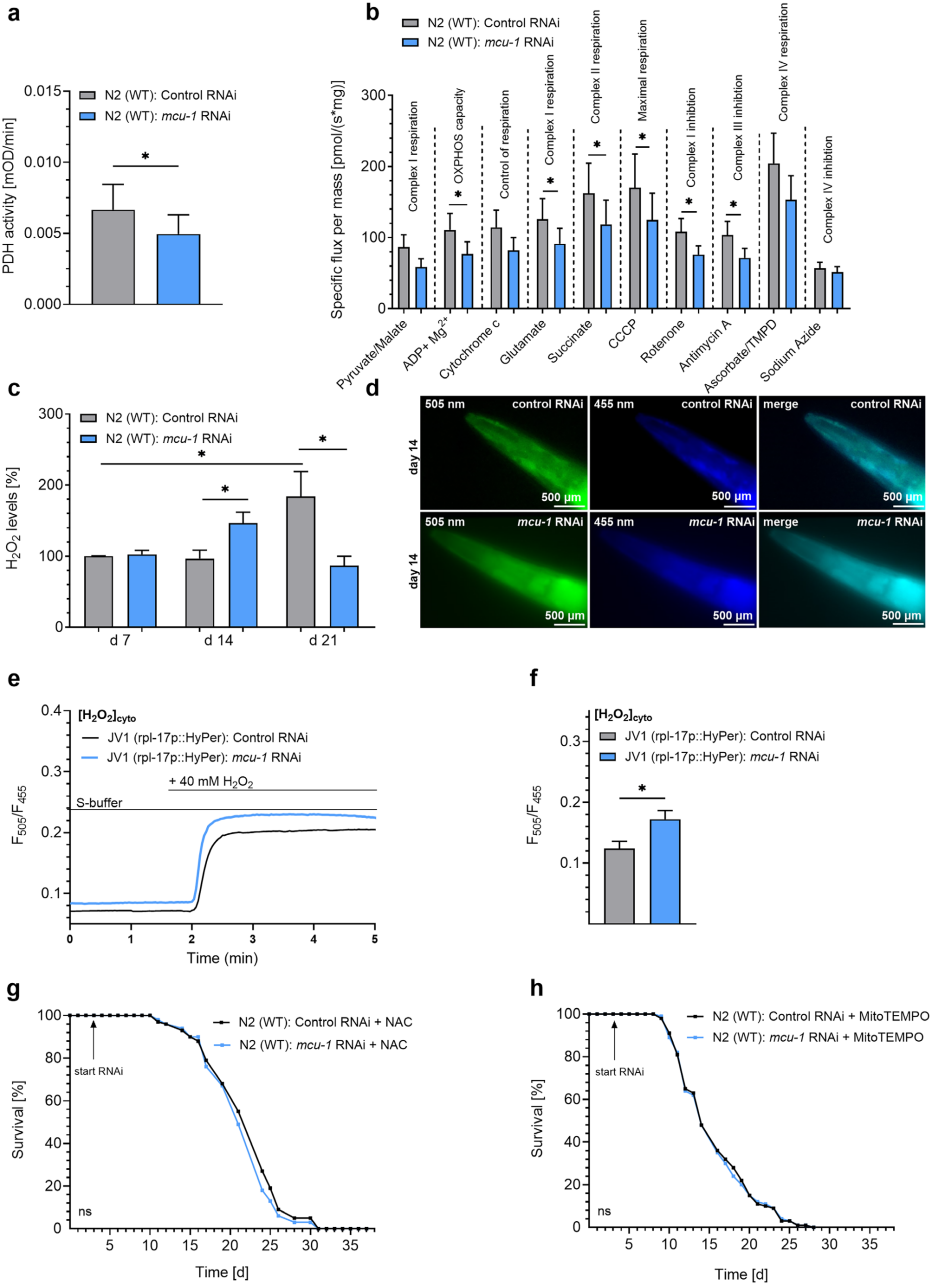
Knockdown of *mcu‐1* modulates TCA, ETC, and H_2_O_2_ levels. (a) Bar graphs (mean ± SEM) represent levels of PDH activity per min in isolated mitochondria of N2 nematodes on day 7, treated with control RNAi (L4440) (gray) or *mcu‐1* RNAi (blue) (*n* = 6). (b) Bar graphs (mean ± SEM) show the results of respirometry of isolated mitochondria of N2 nematodes on day 7, treated with control RNAi (L4440) (gray) or *mcu‐1* RNAi (blue). Vertical lines indicate additions of compounds, including pyruvate [5 mM] + malate [2 mM], adenosine diphosphate (ADP) + Mg^2+^ [2.5 mM], cytochrome c [10 μM], glutamate [10 mM], succinate [10 mM], CCCP [0.5 μM], rotenone [0.5 μM], antimycin A [2.5 μM], ascorbate [2 mM] + TMPD [0.5 mM], and NaN3 [≥ 100 mM] (*n* = 6). (c) Bar graphs (mean ± SEM) represent H_2_O_2_ levels measured by Amplex Red assay in the N2 *C. elegans* strain on days 7, 14, or 21, treated with control RNAi (gray) or *mcu‐1* RNAi (blue) (*n* = 5). (d) Live‐cell imaging of the pharynx in the JV1 *C. elegans* strain expressing the HyPer sensor under a ribosomal promoter, showing emission at 500–521 nm following excitation at either 505 nm (left) or 455 nm (middle) or in the merge (right). Images are presented after treatment with control RNAi (top panels) or RNAi targeting *mcu‐1* (bottom panels). (e) Representative curves showing cytosolic H_2_O_2_ levels in nematodes treated with control RNAi or RNAi against *mcu‐1* expressing HyPer under basal conditions and after the addition of H_2_O_2_ [40 mM]. (f) Bar graphs (mean ± SEM) represent H_2_O_2_ levels in JV1 nematodes on day 14 following treatment with control RNAi (gray) or *mcu‐1* RNAi (blue) (*n* = 5). (g) Lifespan analysis of N2 nematodes treated with control RNAi (black) or *mcu‐1* RNAi (blue) in the presence of 1 mM NAC (*n* = 3). (h) Lifespan analyses of N2 nematodes in the presence of control RNAi (black) and *mcu‐1* RNAi (blue) in the presence of 100 nM MitoTEMPO (*n* = 3). RNAi treatments are indicated by arrows labeled “start RNAi” and, when applicable, “stop RNAi” to mark the beginning and end of the treatment period. N‐numbers are presented as biological replicates. For details, see Table 1. For comparing significant distributions between different groups in the lifespan assays, statistical calculations were carried out using the log‐rank test. If applicable, significant differences were assessed via one‐way ANOVA or unpaired *t*‐test and presented as specific *p* values (*p ≤ 0.05).

Since mitochondrial Ca^2+^ is crucial for the activity of TCA cycle dehydrogenases (Denton and McCormack 1986) and, thus, the activity of the ETC (Mitchell 1961), we assessed the activities of the pyruvate dehydrogenase (PDH) and ETC complexes. In response to *mcu‐1* RNAi treatment, we found a significant reduction in PDH activity (Figure 2a). Sequential substrate and inhibitor titrations further revealed markedly decreased activities of complexes I, II, and III, along with reduced maximal respiration (Figure 2b). Such impairments in ETC function can promote electron leakage and elevated ROS production (Zorov et al. 2014). Therefore, we performed an Amplex Red assay. H_2_O_2_ levels increased progressively from day 7 to day 21 in N2 nematodes. Notably, *mcu‐1* RNAi treatment led to a significant rise in H_2_O_2_ at day 14, followed by reduced levels in late life (day 21) compared to controls (Figure 2c). To further investigate alterations in cytosolic H_2_O_2_ levels, live‐cell imaging was performed in the JV1 strain treated with control RNAi or RNAi against *mcu‐1* on day 14. This strain expresses the H_2_O_2_ sensor HyPer under a ribosomal promoter and was excited at both 455 nm and 505 nm, while excitation was acquired between 500 and 521 nm (Figure 2d). The respective strain was used to assess basal cytosolic H_2_O_2_ levels and the response after H_2_O_2_ application in living nematodes at day 14 under control conditions or with *mcu‐1* knockdown (Figure 2e). This approach confirmed the transient increase in cytosolic H_2_O_2_ levels at day 14 (Figure 2f). Consequently, we further investigated whether alterations in H_2_O_2_ levels are associated with lifespan extension induced by *mcu‐1* RNAi. Treatment with 1 mM of the ROS scavenger N‐acetyl cysteine (NAC) (Schulz et al. 2007) abolished the lifespan‐extending effects of *mcu‐1* RNAi in N2 nematodes (Figure 2g and Table 1), as did the mitochondrial‐specific antioxidant MitoTEMPO [100 nM] (Ewald et al. 2017) (Figure 2h; Table 1). These results suggest that a transient increase in ROS levels may trigger a signaling cascade that promotes longevity and improves fitness in older worms.

### 2.3 Reduced Mitochondrial Ca^2+^ Levels Induce Lifespan Extension Through a ROS‐Activated Signaling Pathway

**FIGURE 3:**
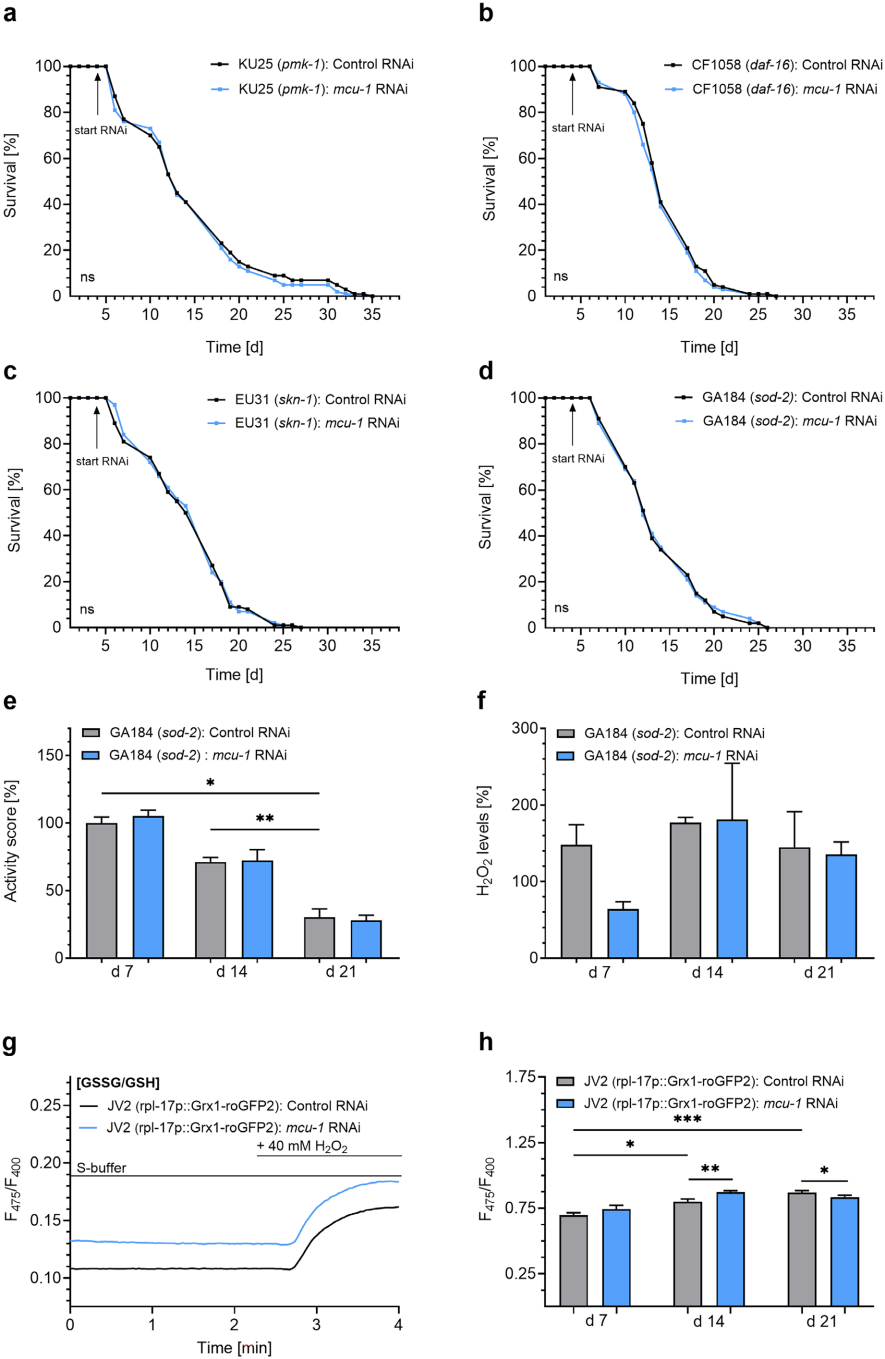
The ROS‐mediated signaling cascade triggered by *mcu‐1* RNAi involves *pmk‐1, daf‐16*, and *skn‐1*. (a) Lifespan analyses of the KU25 (*pmk‐1*/km25 deficiency) *C. elegans* strain in the presence of control RNAi (L4440) (black) or *mcu‐1* RNAi (blue) (*n* = 3). (b) Lifespan analyses of the CF1038 (*daf‐16*/mu86 deficient) *C. elegans* strain in the presence of control RNAi (black) or *mcu‐1* RNAi (blue) (*n* = 3). (c) Lifespan analyses of the EU31 (*skn‐1*/zu135) *C. elegans* strain in the presence of control RNAi (black) or *mcu‐1* RNAi (blue) (*n* = 3). (d) Lifespan analyses of the GA184 (*sod‐2*/gk257 deficiency) *C. elegans* strain in the presence of control RNAi (black) or *mcu‐1* RNAi (blue) (*n* = 3). RNAi treatments are indicated by arrows labeled “start RNAi” and, when applicable, “stop RNAi” to mark the beginning and end of the treatment period. (e) Bar graphs (mean ± SEM) represent activity score (%), based on body bends per min, in the GA184 (*sod‐2*/gk257 deficiency) *C. elegans* strain on day 7, 14, and 21 following the treatment with control RNAi (L4440) (gray) or *mcu‐1* RNAi (blue) (*n* = 3). (f) Bar graphs (mean ± SEM) represent H_2_O_2_ levels measured by Amplex Red assay in the GA184 (*sod‐2*/gk257 deficiency) *C. elegans* strain on days 7, 14, or 21, treated with control RNAi (gray) or *mcu‐1* RNAi (blue). (g) Representative curves showing the oxidized/reduced Grx1‐roGFP2 levels [GSSG/GSH] in the JV2 *C. elegans* strain, after treatment with control RNAi (black) or RNAi against *mcu‐1* (blue), under basal conditions and after the addition of H_2_O_2_ [40 mM]. (h) Bar graphs (mean ± SEM) represent oxidized/reduced GRX‐1‐roGFP2 levels in the JV2 *C. elegans* strain on days 7, 14, and 21, treated with control RNAi (gray) or *mcu‐1* RNAi (blue) (*n* = 4). N‐numbers are presented as biological replicates. For details, see Table 1. For comparing significant distributions between different groups in the lifespan assays, statistical calculations were carried out using the log‐rank test. If applicable, significant differences were assessed via one‐way ANOVA or unpaired *t*‐test and presented as specific *p* values (*p ≤ 0.05, **p ≤ 0.01, ***p ≤ 0.001).

To investigate whether lifespan extension caused by *mcu‐1* knockdown is dependent on ROS‐sensitive signaling pathways, we tested worms deficient in *pmk‐1, daf‐16*, and *skn‐1*, the respective nematode orthologs of human p38 mitogen‐activated protein kinases (MAPK) (Inoue et al. 2005), forkhead box transcription factor class O (FOXO), and nuclear factor erythroid 2‐related factor 2 (NRF2) (Tian et al. 2021). In *pmk‐1*‐deficient worms, *mcu‐1* RNAi failed to extend lifespan (Figure 3a; Table 1), as it also did in worms deficient in *daf‐16* (Figure 3b; Table 1) and *skn‐1* (Figure 3c; Table 1). Notably, deficiency in AMP‐activated protein kinase (AMPK) (*aak‐2*) or sirtuins (*sir‐2.1*) could not prevent lifespan extension induced by *mcu‐1* RNAi (Figure S2), suggesting that the effect is mediated by a signaling cascade triggered by transient ROS fluctuations rather than by nutrient‐sensing pathways. Since DAF‐16 and SKN‐1 promote antioxidant defense mechanisms, we also tested worms deficient in superoxide dismutase (*sod‐2*). Results from respective experiments demonstrate that both the lifespan‐extending (Figure 3d; Table 1) and fitness‐enhancing (Figure 3e) effects of *mcu‐1* RNAi were abolished in *sod‐2* deficient worms, which did not exhibit the characteristic H_2_O_2_ rise as seen in N2 nematodes during aging or in response to *mcu‐1* RNAi at day 14 (Figure 3f). Next, the JV2 strain (Back et al. 2012) was used to assess the basal ratio of oxidized to reduced glutathione (GSSG/GSH) levels, before and after the addition of H_2_O_2_ in nematodes with and without knockdown of *mcu‐1* (Figure 3g). During aging, the GSSG/GSH ratio increased in JV2 nematodes. Notably, GSSG levels increased during middle age in nematodes treated with *mcu‐1* RNAi, while a reduced GSSG/GSH ratio was observed in old age in treated worms (Figure 3h). In parallel, we examined the expression of two established DNA damage markers, the programmed cell death activator *egl‐1* and cell death protein 3 subunit *p17* (*ced‐3*) (Greiss et al. 2008), which showed a tendency to be upregulated with age but were significantly downregulated at day 21 in worms treated with *mcu‐1* RNAi, supporting the notion that ROS‐induced signaling pathways involving FOXO and NRF2 counteract age‐associated genotoxic stress (Figure S2).

In summary, these results indicate a shift in ROS homeostasis through mitochondrial Ca^2+^ modulation, involving *pmk‐1*, *daf‐16*, and *skn‐1*, which lead to enhanced antioxidant defense mechanisms and lifespan extension following *mcu‐1* RNAi treatment.

### 2.4 Reduced Mitochondrial Ca^2+^ Levels Normalize Mitochondrial Structure and Function in Aged Nematodes

**FIGURE 4:**
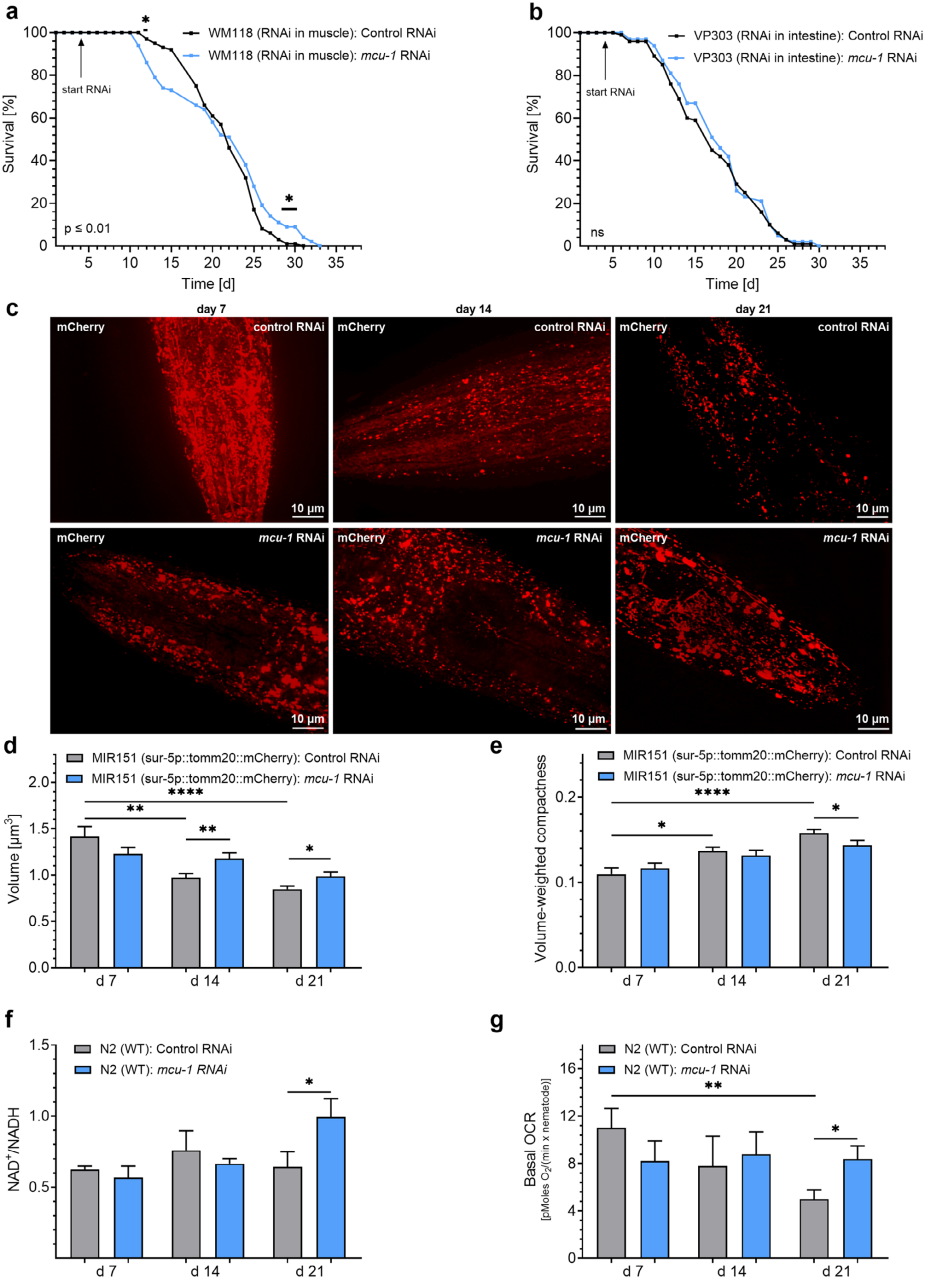
RNAi‐induced *mcu‐1* knockdown normalizes mitochondrial structure and function in aged nematodes. (a) Lifespan analyses of the WM118 *C. elegans* strain, allowing muscle‐specific RNAi knockdown, in the presence of control RNAi (L4440) (black) or *mcu‐1* RNAi (blue) (*n* = 3). (b) Lifespan analyses of the VP303 *C. elegans* strain, allowing intestine‐specific RNAi knockdown, in the presence of control RNAi (L4440) (black) or *mcu‐1* RNAi (blue) (*n* = 3). RNAi treatments are indicated by arrows labeled “start RNAi” and, when applicable, “stop RNAi” to mark the beginning and end of the treatment period. (c) Confocal microscopy picture showing the emission at 540 nm in nematodes of the MIR151 strain, expressing mCherry sensor fused to the mitochondrion targeting sequence (tomm‐20(AA1‐55)) under the ubiquitous sur‐5 promoter, after treatment with control RNAi (top panels) or RNAi against *mcu‐1* (bottom panels) on days 7 (left), 14 (middle), and 21 (right). Bar graphs represent (d) mitochondrial volume [μm^3^] and (e) volume‐weighted compactness in the MIR151 *C. elegans* strain on days 7, 14, and 21, treated with control RNAi (gray) or *mcu‐1* RNAi (blue) (*n* = 4). (f) Bar graphs represent NAD^+^/NADH + H^+^ levels in N2 nematodes on days 7, 14, and 21, treated with control RNAi (gray) or *mcu‐1* RNAi (blue) (*n* = 3). (g) Bar graphs show basal OCR assessed by the Seahorse XF Cell Mito stress assay in N2 nematodes on days 7, 14, and 21, treated with control RNAi (gray) or *mcu‐1* RNAi (blue) (*n* = 3). *N* numbers are presented as biological replicates. For details, see Table 1. For comparing significant distributions between different groups in the lifespan assays, statistical calculations were carried out using the log‐rank test. If applicable, significant differences were assessed via one‐way ANOVA or unpaired *t*‐test and presented as specific *p* values (*p ≤ 0.05, **p ≤ 0.01, ****p ≤ 0.0001).

Next, we utilized the W118 strain to test whether muscle‐specific knockdown of *mcu‐1* is sufficient to extend lifespan. Indeed, overall lifespan was found to be significantly increased by *mcu‐1* RNAi in this strain (Figure 4a, Table 1), while no lifespan extension was found in the VP303 strain with specific *mcu‐1* knockdown in the intestine (Figure 4b, Table 1). Since muscle function is strongly relying on mitochondrial function (Gan et al. 2018; Wu et al. 2023) and NRF2 activation has been linked to increased mitochondrial biogenesis (Kraft et al. 2006), we also investigated mitochondrial structure by confocal microscopy throughout aging and in response to *mcu‐1* RNAi in the newly developed worm strain MIR151 expressing mCherry under the sur‐5 promotor in the mitochondrial matrix (Figure 4c). Mitochondrial volume (Figure 4d) and surface area (Figure S3) declined, while volume‐weighted compactness (Figure 4e) and volume‐weighted sphericity (Figure S3) increased during aging, indicating a shift toward smaller, more spherical mitochondria consistent with progressive mitochondrial fragmentation during aging. Knockdown of *mcu‐1* mitigated these age‐related alterations, restoring normal mitochondrial morphology by day 21 (Figures 4d,e and S3). Notably, treatment with *mcu‐1* RNAi mitigated these age‐associated structural changes, maintaining larger and more interconnected mitochondria at both day 14 and day 21 (Figures 4c and S3). Functionally, *mcu‐1* RNAi‐treated N2 worms showed an improved NAD^+^/NADH ratio at day 21 (Figure 4f). Moreover, *mcu‐1* RNAi caused an increase in basal oxygen consumption rate (OCR) in N2 nematodes at day 21, counteracting the age‐related decrease in OCR (Figure 4g).

These data suggest that reduced mitochondrial Ca^2+^ uptake not only preserves mitochondrial structure but also enhances mitochondrial function in late adulthood.

### 2.5 Pharmacological Inhibition of the MCU Complex Mimics *MCU‐1* Knockdown in Nematodes

**FIGURE 5:**
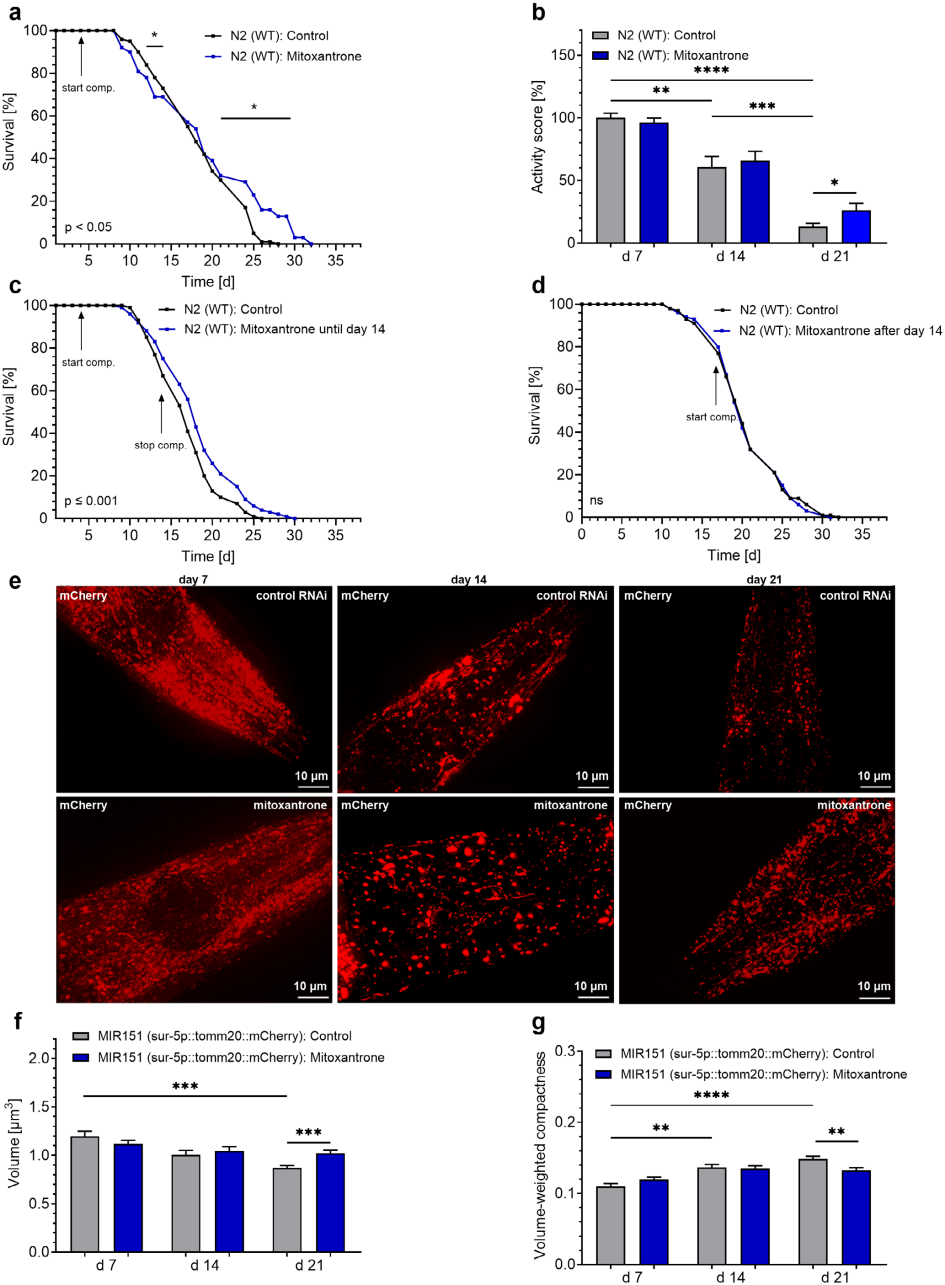
Pharmacological inhibition of MCU‐1 mimics the effects of *mcu‐1* RNAi in nematodes. Lifespan analyses of N2 nematodes in the presence of DMSO (0.001%) (black) or mitoxantrone (10 nM) (blue) (*n* = 3). (b) Bar graphs (mean ± SEM) represent activity score (%), based on body bends per min, in N2 nematodes on days 7, 14, and 21 following the treatment with DMSO (0.001%) (black) or mitoxantrone (10 nM) (blue) (*n* = 3). (c) Lifespan analyses of N2 nematodes treated with DMSO (0.001%) (black) or mitoxantrone (10 nM) (blue) until day 14 (*n* = 3). (d) Lifespan analyses of N2 nematodes treated with DMSO (0.001%) (black) or mitoxantrone (10 nM) (blue) after day 14 (*n* = 3). Compound treatments are indicated by arrows labeled “start comp” and, when applicable, “stop comp” to mark the beginning and end of the treatment. (e) Confocal microscopy picture showing the emission at 540 nm in nematodes of the MIR151 strain, expressing mCherry sensor fused to the mitochondrion targeting sequence (tomm‐20(AA1‐55)) under ubiquitous sur‐5 promoter, after treatment with DMSO (0.001%) (top panels) or mitoxantrone (10 nM) (bottom panels) on days 7 (left), 14 (middle), and 21 (right). Bar graphs represent (f) mitochondrial volume (μm^3^) and (g) volume‐weighted compactness in the MIR151 *C. elegans* strain on days 7, 14, and 21, treated with DMSO (0.001%) (gray) or mitoxantrone (10 nM) (blue) (*n* = 3). N‐numbers are presented as biological replicates. For details, see Table 1. For comparing significant distributions between different groups in the lifespan assays, statistical calculations were carried out using the log‐rank test. If applicable, significant differences were assessed via one‐way ANOVA or unpaired *t*‐test and presented as specific *p* values (*p ≤ 0.05, **p ≤ 0.01, ***p ≤ 0.001, ****p ≤ 0.0001).

Given the FDA's approval for the use of the MCU inhibitor mitoxantrone in humans (Fox 2004), we used this compound to test whether a low dosage of mitoxantrone could replicate the effects of *mcu‐1* RNAi. Treatment with 10 nM mitoxantrone reduced lifespan during middle age significantly (days 12–14) but extended the lifespan of surviving N2 worms (Figure 5a, Table 1) and improved motility in aged nematodes at day 21 (Figure 5b, Table 1), similar to the effects observed with *mcu‐1* RNAi. Like *mcu‐1* RNAi, mitoxantrone significantly extended the N2 nematodes' lifespan when administered until day 14 (Figure 5c, Table 1) but was ineffective when applied after day 14 (Figure 5d, Table 1). As for *mcu‐1* knockdown worms, confocal microscopy of the MIR151 strain treated with dimethyl sulfoxide (DMSO) or mitoxantrone revealed that mitoxantrone increased mitochondrial volume (Figure 5f) and surface area (Figure S4), while decreasing volume‐weighted compactness (Figure 5g) and volume‐weighted sphericity (Figure S4) on day 21, indicating a shift toward more elongated and less spherical mitochondrial morphology.

These findings indicate that the FDA‐approved drug mitoxantrone, at low doses, may mimic the effects of *mcu‐1* RNAi.

### 2.6 Reduced Mitochondrial Ca^2+^ Uptake Induces Transient Oxidative Stress and Antioxidant Adaptation in HFF‐1 Cells

**FIGURE 6:**
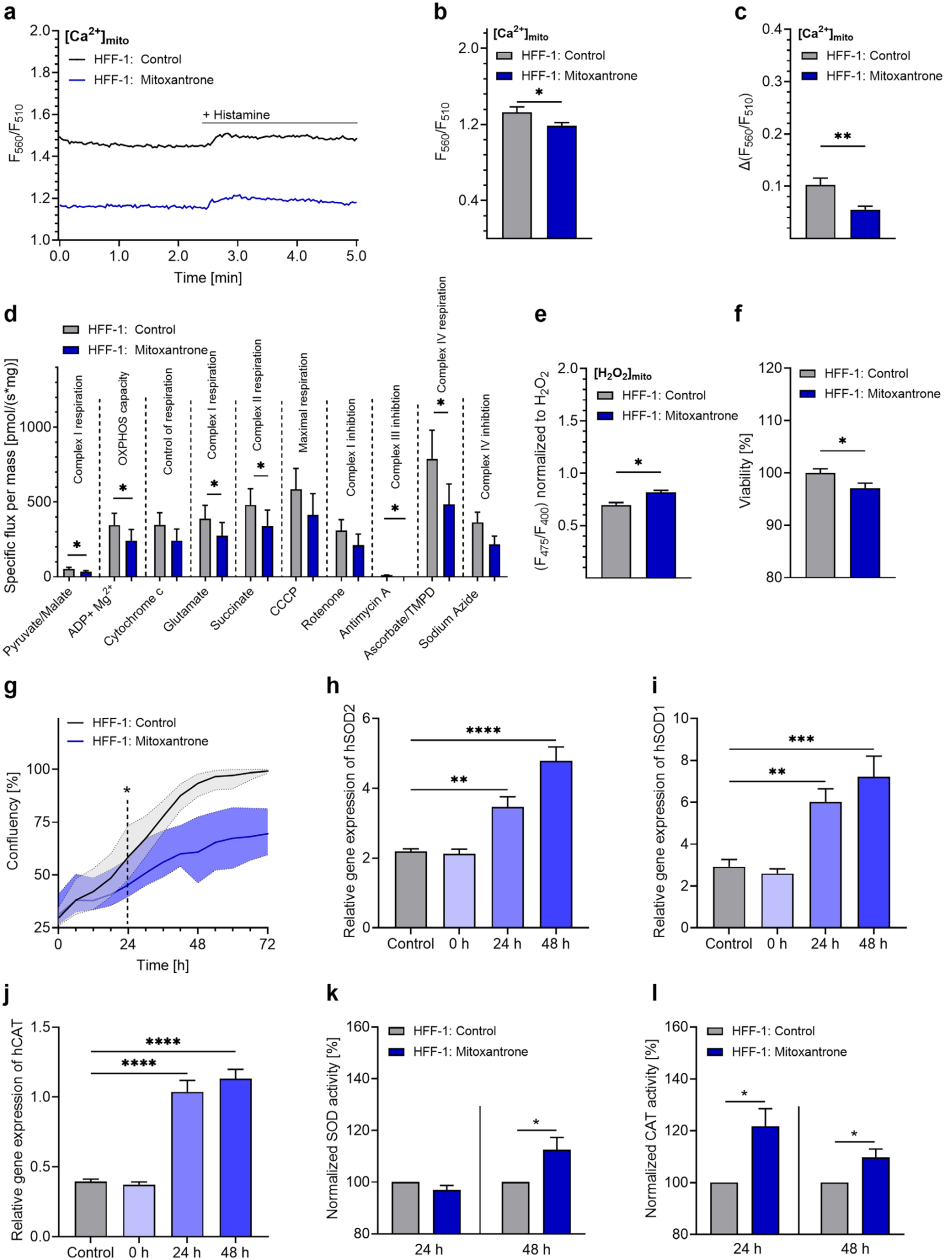
Mitoxantrone‐induced mitochondrial Ca^2+^ dysregulation triggers adaptive antioxidant responses in HFF‐1 cells. Representative curves showing [Ca^2+^]_mito_ levels in HFF‐1 cells expressing mtD1GO treated with DMSO (0.001%) (black) or mitoxantrone (100 nM) (blue) for 3 h, under basal conditions and after application of 100 μM histamine. (b) Bar graphs represent basal [Ca^2+^]_mito_ levels in HFF‐1 cells treated with DMSO (0.001%) (control) or mitoxantrone (control *n* = 25/3; mitoxantrone *n* = 30/3). (c) Bar graphs represent [Ca^2+^]_mito_ uptake in HFF‐1 cells treated with DMSO (0.001%) (control) or mitoxantrone (control *n* = 25/3; mitoxantrone *n* = 30/3). (d) Bar graphs (mean ± SEM) show specific flux per mass in isolated mitochondria of HFF‐1 cells treated with DMSO (0.001%) (gray) or mitoxantrone (100 nM) (blue) for 3 h. Vertical lines indicate additions of compounds, including pyruvate (5 mM) + malate (2 mM), adenosine diphosphate (ADP) + Mg^2+^ (2.5 mM), cytochrome c (10 μM), glutamate (10 mM), succinate (10 mM), CCCP (0.5 μM), rotenone (0.5 μM), antimycin A (2.5 μM), ascorbate (2 mM) + TMPD (0.5 mM), and NaN_3_ (≥ 100 mM). (e) Bar graphs (mean ± SEM) represent mitochondrial H_2_O_2_ production of HFF‐1 cells expressing HyPer in mitochondria, treated with DMSO (0.001%) (control) or mitoxantrone (100 nM) (control *n* = 43/4; mitoxantrone *n* = 40/4). (f) Bar graphs (mean ± SEM) show cell viability of HFF‐1 cells 24 h after treatment with DMSO (0.001%) (gray) or mitoxantrone (100 nM) (blue) (*n* = 4). (g) Proliferation rates (mean ± range) presented as percentage of confluency of cells within 72 h after treatment with DMSO (0.001%) (gray) or mitoxantrone (100 nM) (blue) (*n* = 5). Bar graphs (mean ± SEM) represent the relative expression levels of (h) SOD2, (i) SOD1, and (j) CAT in HFF‐1 cells before and 0 h, 24 h, and 48 h after treatment with DMSO (0.001%) (gray) and mitoxantrone (100 nM) (blue) (*n* = 4). Bar graphs represent enzymatic activity levels of (k) SODs and (l) CAT 24 h and 48 h after treatment with DMSO (0.001%) (gray) and mitoxantrone (100 nM) (blue) (*n* = 4). If applicable, significant differences were assessed via unpaired *t*‐test and presented as specific *p* values (*p ≤ 0.05, **p ≤ 0.01, ***p ≤ 0.001, ****p ≤ 0.0001).

To assess the translatability of our findings from nematodes to mammalian cells, we measured organellar Ca^2+^ and H_2_O_2_ levels in HFF‐1 cells following short‐term (3 h) treatment with a low dose [100 nM] of mitoxantrone. Live‐cell imaging of HFF‐1 cells expressing the mitochondrial Ca^2+^ sensor mtD1GO (Figure 6a) demonstrated significantly reduced basal mitochondrial Ca^2+^ levels (Figure 6b) and decreased mitochondrial Ca^2+^ uptake in response to 100 μM histamine (Figure 6c) after 3 h of mitoxantrone treatment. As in nematodes, reduced mitochondrial Ca^2+^ uptake in HFF‐1 cells was accompanied by a significant reduction in the activity of complex I, II, and III, as well as of complex IV (Figures 6d and S5). Live‐cell imaging utilizing the mitochondrial Hyper7 sensor also revealed increased mitochondrial H_2_O_2_ levels in HFF‐1 cells after 3 h of mitoxantrone [100 nM] treatment (Figure 6e). Similar to findings in nematodes, mitoxantrone treatment induced a transient decline in cell survival, with significantly reduced viability observed 24 h after the 3 h treatment with 100 nM mitoxantrone (Figure 6f). Proliferation analyses revealed that although many cells underwent sustained growth arrest due to mitoxantrone treatment, a subset continued to divide (Figures 6g and S5). This heterogeneous response may reflect a limited adaptive capacity that enables survival and partial regrowth within the more resistant cell fraction. As a *mitohormetic* response is typically characterized by enhanced antioxidant defenses following transient stress, we next assessed the expression and activity of key antioxidant enzymes after exposure to mitoxantrone. Notably, mRNA levels of SOD2 (Figure 6h), SOD1 (Figure 6i), and CAT (Figure 6j) were significantly elevated at 24 h and increased further at 48 h posttreatment. Consistently, the enzymatic activity of SODs was significantly elevated only at 48 h (Figure 6k), whereas CAT activity increased as early as 24 h and remained elevated at 48 h (Figure 6l).

These findings indicate that impaired mitochondrial Ca^2+^ uptake triggers a transient rise in ROS levels, followed by an adaptive response characterized by upregulation of the cellular antioxidant defense system. Consequently, targeting mitochondrial Ca^2+^ uptake may represent a promising therapeutic strategy to enhance health and lifespan.

## 3 Discussion

Our findings demonstrate that reducing mitochondrial Ca^2+^ uptake modestly via *mcu‐1* RNAi extends lifespan and enhances motility in aged nematodes, despite impairing survival during early adulthood. This lifespan extension is linked to a transient increase in mitochondrial H_2_O_2_ levels, which initiates a signaling cascade involving *pmk‐1* (p38 MAPK), *daf‐16* (FOXO), and *skn‐1* (NRF2). This pathway enhances antioxidant defense, mitigates oxidative stress, and preserves mitochondrial integrity during the aging process. Importantly, pharmacological inhibition of mitochondrial Ca^2+^ uptake with mitoxantrone mirrored the beneficial effects of *mcu‐1* RNAi in nematodes and human HFF‐1 cells, further demonstrating the promising therapeutic potential of targeting mitochondrial Ca^2+^ regulation in aging and possibly in age‐related diseases.

A previous study using the *mcu‐1(ju1154) mutant* strain demonstrated that stable *mcu‐1* deficiency extends lifespan and enhances motility up to day 13. This report suggested that inhibiting MCU‐1 function preserves muscle health during aging by mitigating age‐related mitochondrial Ca^2+^ accumulation and subsequent activation of mitophagy (Higashitani et al. 2023). While our experiments confirmed an age‐related mitochondrial Ca^2+^ increase, even until day 21 (Figure 1g), we observed a biphasic response to *mcu‐1* RNAi during the lifespan of *C. elegans* , with reduced survival during middle age and extended lifespan (Figure 1a), accompanied by increased motility during old age (Figure 1b). Notably, we found that hampering mitochondrial Ca^2+^ uptake until day 14 is sufficient to provoke longevity (Figure 1c), while *mcu‐1* RNAi treatment initiated only in old age failed to extend lifespan (Figure 1d). These results underscore the importance of adaptational mechanisms activated during middle age by transient *mcu‐1* knockdown in driving longevity. The previously used *mcu‐1(ju1154) mutant* may have already acquired these mechanisms related to ROS defense due to long‐term adaptations, which could have contributed to enhanced motility during middle age (Higashitani et al. 2023).

Our data also revealed that mitochondrial Ca^2+^ uptake is associated with decreased activity of the PDH (Figure 2a) and the ETC (Figure 2b), which is the key site of ROS production (Madreiter‐Sokolowski et al. 2020). To obtain the large number of nematodes required for mitochondrial isolation in these assays, we switched from plate culture to liquid culture. We acknowledge that liquid culture conditions can introduce physiological differences (Hibshman et al. 2021; Laranjeiro et al. 2017). Therefore, all comparisons were made against control samples grown under the same liquid culture conditions to assess accurately the effects of impaired mitochondrial Ca^2+^ uptake.

While the exact species of ROS induced by mitochondrial Ca^2+^ uptake inhibition remains elusive due to a lack of appropriate methods (Griendling et al. 2016), a transient rise in H_2_O_2_ has been found in middle‐aged worms treated with *mcu‐1* RNAi by Amplex Red assay (Figure 2c) and live‐cell imaging (Figure 2f). Prevention of *mcu‐1* RNAi‐induced lifespan extension by the global ROS scavenger NAC (Figure 2g) and the mitochondria‐specific antioxidant MitoTEMPO (Figure 2h) further highlighted the crucial role of ROS in the lifespan extension provoked by mitochondrial Ca^2+^ uptake inhibition. Notably, NAC and MitoTEMPO scavenge different types of ROS. While NAC primarily scavenges H_2_O_2_ and hydroxyl radicals (Aruoma et al. 1989), MitoTEMPO specifically scavenges mitochondrial superoxide anions (McCarthy and Kenny 2016). Consequently, these findings suggest that multiple types of ROS may contribute to the lifespan extension induced by mitochondrial Ca^2+^ uptake inhibition. Given that H_2_O_2_ is the primary activator of p38 MAPK (Angeloni et al. 2011), other ROS likely influence p38 MAPK indirectly through their conversion to H_2_O_2_.

Over the past decade, the so‐called *mitohormetic* signaling, induced by transient ROS increases and linked to enhanced antioxidant defenses, has emerged as a key driver of longevity in different organisms (Ristow and Schmeisser 2014). In line with previously reported ROS‐induced signaling cascades, mitochondrial Ca^2+^ uptake inhibition‐induced longevity was also related to *pmk‐1* (p38 MAPK) (Figure 3a) and the transcription factors *daf‐16* (FOXO) (Figure 3b) and *skn‐1* (NRF2) (Figure 3c), which were associated with enhanced antioxidant enzyme activity like *sod‐2* (Figure 3d–f). Previous results also found that the induction of AMPK (*aak‐2*) and sirtuins (*sir‐2.1*) by inhibiting the ETC (Tian et al. 2021) is an essential part of ROS‐induced longevity signaling. Interestingly, lifespan extension induced by *mcu‐1* knockdown was observed even in worms deficient in AMPK (*aak‐2*) or sirtuins (*sir‐2.1*) (Figure S2), suggesting that mitochondrial Ca^2+^ uptake inhibition does not increase cytosolic levels of AMP and NAD^+^ necessary to activate AMPK and sirtuins.

While ROS levels increased at day 14 in *mcu‐1* knockdown nematodes, genes associated with DNA damage were not upregulated (Figure S2), suggesting that activation of the DNA damage response is not the primary cause of the observed reduction in early‐life survival (Figure 1a). We propose that the transient decrease in survival is more likely due to short‐term mitochondrial dysfunction, possibly involving redox imbalance and oxidation of critical proteins, rather than genotoxic stress. Interestingly, by day 21, DNA damage response genes were consistently downregulated in *mcu‐1* knockdown nematodes compared to controls (Figure S2), potentially indicating improved genomic stability later in life. This may result from enhanced cellular stress resistance, in line with the concept of *mitohormesis*.

Confocal microscopy revealed that *mcu‐1* RNAi (Figures 4c,e and S3), as well as mitoxantrone treatment (Figures 5e–g and S4), reverses age‐associated mitochondrial fragmentation, which was in line with enhanced mitochondrial functional activity in *mcu‐1* knockdown nematodes (Figure 4f,g). These data align with previous findings suggesting improved mitochondrial structure due to *mcu‐1* deficiency. However, as mentioned above, the beneficial effects were observed at an earlier stage in the *mcu‐1* deficient strain (Higashitani et al. 2023), potentially due to previously acquired adaptive mechanisms.

The availability of compounds modulating mitochondrial Ca^2+^ uptake, including several substances targeting MCU (Wang et al. 2025) and MICU1 (Di Marco et al. 2020), underscores the feasibility of pharmacological interventions targeting mitochondrial Ca^2+^ homeostasis in humans. Mitoxantrone, for example, is even FDA‐approved for the treatment of specific cancer types and multiple sclerosis (Fox 2004). Nevertheless, its potential adverse effects necessitate careful evaluation before considering any application beyond established indications, such as in the context of aging research. Our findings suggest that mitoxantrone's potential as an antiaging drug may rely on much lower concentrations than those used in cancer therapy, which could also reduce potential side effects. A concentration of 10 nM mitoxantrone mimicked the effects of *mcu‐1* RNAi in nematodes, causing a biphasic lifespan response (Figure 5a), enhanced motility in old age (Figure 5b), early‐age‐specific lifespan effects not observed with treatment initiated in old age (Figure 5c,d), as well as preservation of mitochondrial structure during old age (Figures 5e–g and S4). Based on previously reported results, 0.2 μM mitoxantrone has been shown to impair the proliferation of human cancer cells (Almeida et al. 2018), and a concentration of 10 μM has achieved half‐maximal inhibition of mitochondrial Ca^2+^ uptake (Arduino et al. 2017). However, our experiments showed that treating noncancerous HFF‐1 for 3 h with 100 nM mitoxantrone was sufficient to reduce basal mitochondrial Ca^2+^ levels and uptake (Figure 6a–c), block ETC complexes (Figure 6d), and increase ROS levels (Figure 6e). The temporal pattern observed in subsequent viability assays and experiments to assess antioxidant defense mechanisms suggests a heterogeneous cellular response to mitoxantrone‐induced stress. The marked decline in cell viability at 24 h (Figure 6f) likely reflects acute cytotoxic effects due to oxidative stress. While a substantial fraction of cells appeared to undergo sustained growth arrest, some cells retained limited proliferative potential (Figures 6g and S5). This effect is consistent with the dual pharmacological profile of mitoxantrone, which, in addition to inhibiting MCU activity, acts as a potent topoisomerase II inhibitor and DNA intercalator, leading to cell cycle arrest (Evison et al. 2016). The delayed upregulation of antioxidant defense enzymes (Figure 6h–j) may therefore represent an adaptive response in the more resistant cells, aimed at restoring redox balance and enabling partial regrowth despite the overall adverse impact of mitoxantrone. Interestingly, transcriptional induction of SOD2 (Figure 6h), SOD1 (Figure 6i), and CAT (Figure 6j) mRNA was already evident at 24 h, yet enzymatic activity followed distinct kinetics. SOD activity increased only after 48 h (Figure 6k), suggesting a time lag between transcriptional activation, protein synthesis, and maturation of functional enzymes. In contrast, CAT activity was significantly elevated as early as 24 h after treatment (Figure 6l), indicating that catalase may undergo rapid posttranslational activation. Given catalase's primary role in decomposing H_2_O_2_, its early activation could reflect an immediate need to detoxify excess H_2_O_2_ generated during the acute oxidative stress phase, whereas SOD activation becomes more prominent later to mitigate superoxide accumulation during recovery.

Importantly, the discrepancy between the beneficial effects observed in *C. elegans* and the partly adverse outcomes in human fibroblasts may, at least in part, also reflect the proliferative status of the cells: nematode somatic cells are largely postmitotic, whereas HFF‐1 cells are proliferative. Thus, mitoxantrone may exert more favorable effects in postmitotic contexts by modulating mitochondrial Ca^2+^ handling, while in proliferating cells, the predominant outcome for a significant fraction of cells is growth arrest due to topoisomerase II inhibition (Evison et al. 2016).

To effectively induce a *mitohormetic* response without side effects by inhibiting mitochondrial Ca^2+^ uptake in human cells, both the specificity of the compound and the timing of its application need to be carefully optimized. Specifically, compounds with minimal off‐target effects that selectively inhibit MCU are preferable. The cytotoxic effects of mitoxantrone due to its ability to inhibit topoisomerase II (Evison et al. 2016), for instance, limit its suitability as an antiaging compound that modulates mitochondrial Ca^2+^ handling. This underscores the need for identifying more specific MCU‐targeting agents—potentially derivatives of Ruthenium Red or novel small‐molecule inhibitors of the mitochondrial Ca^2+^ uptake machinery (Marta et al. 2021).

In summary, this study reveals that genetically and pharmacologically decreased mitochondrial Ca^2+^ uptake triggers a ROS‐mediated signaling cascade that preserves mitochondrial function and delays the onset of age‐related mitochondrial deterioration, which results in the longevity and enhanced motility of *C. elegans*. Given the parallels between nematode and mammalian Ca^2+^ and ROS homeostasis, as well as the partial translatability of viability results to human cells, our data highlight the therapeutic relevance of targeting mitochondrial Ca^2+^ uptake for preventing age‐related mitochondrial dysfunction and promoting healthy aging.

## 4 Methods

### 4.1 Nematode Strain and Culture Conditions

*C. elegans* strains were obtained from the Caenorhabditis Genetics Center (CGC, University of Minnesota, US). The strains used in the present study involved N2 wild type (Bristol), KU25 (*pmk‐1*/km25), GA184 (*sod‐2*/gk257), CF1038 (*daf‐16*/mu86), EU31 (*skn‐1*/zu135), Mir248 (*aak‐2*/ok524), Mir14 (*sir‐2.1*/ok434), JV1 (jrIs1 [rpl‐17p::HyPer + unc‐119(+)] stable transgene expressing YFP‐based hydrogen peroxide sensor HyPer under ribosomal promotor) (Back et al. 2012), VP303 (kbIs7 [nhx‐2p::rde‐1 + rol‐6(su1006)]. Rollers. RNAi effective only in intestine), WM118 (neIs9 [myo‐3::HA::rde‐1 + rol‐6(su1006)] X. Transgene rescues muscle RNAi defect. Rollers.), and JV2 (jrIs2 [rpl‐17p::Grx1‐roGFP2 + unc‐119(+)] stable transgene ubiquitously expressing the roGFP2 sensor under a ribosomal promoter for in vivo estimation of GSSG/GSH ratios) (Back et al. 2012).

MIR151 (risIs10[sur‐5p::tomm20(AA1‐55)::mCherry + unc119(+)]) expressing mitochondrial mCherry sensor was generated by microparticle bombardment of strain HT1593 (unc‐119(ed3) III) with a suitable expression vector (Grigolon et al. 2022). Briefly, the expression vector was generated by Gateway cloning using pENTRY‐sur‐5p, pENTRY‐tomm20(AA1‐55)::mCherry, and pDEST‐ZK001 (modified pDEST‐MB14, lacking GFP).

AQ3055 expressing mitochondrial‐targeted YC3.60 sensor as an extrachromosomal array on the pharynx under the myo‐2 promoter (pmyo‐2::2mt8::YC3.60) was a kind gift from Javier Alvarez (Alvarez‐Illera et al. 2017).

Nematodes were grown at 20°C on agar plates with nematode growth media (NGM) with high gel strength agar (SERVA, Germany), which was spotted with *Escherichia coli* ( *E. coli* ), using the OP50 strain to maintain the strains as described previously (Brenner 1974; Schulz et al. 2007).

### 4.2 RNAi and Compound Treatment

Nematodes were treated from day 4 on, after development. RNAi‐induced knockdown was performed as described (Oh et al. 2006). The HT115(DE3) *E. coli* strain transformed with the L4440 vector (empty vector) was used as a control, and the K02B2.3 vector with RNAi targeting *mcu‐1* was used for RNAi treatment (Kamath and Ahringer 2003). Plates were supplemented with 1 mM isopropyl β‐D‐1‐thiogalactopyranoside (IPTG) (Roth, Austria) and ampicillin (100 mg/mL) (Roth, Austria) unless stated otherwise. The RNAi feeding protocol began on day 4 to avoid disrupting developmental processes (De‐Souza et al. 2019).

Compound treatment of nematodes was carried out on NGM agar plates containing mitoxantrone (10 nM) and control plates with the corresponding solvent, 0.001% DMSO (Roth, Austria) (Schmeisser et al. 2011). The antioxidants mitoTEMPO and NAC (Merck KGaA, Germany) were applied at a final concentrations of 100 nM and 1 mM, respectively (Zarse et al. 2012).

### 4.3 Lifespan Analysis

To obtain a synchronized nematode population, worms were allowed to lay eggs for 5 days at 20 C on agar plates and then removed with S‐buffer. Eggs were subsequently transferred to fresh NGM plates. After 4 days, 50 L4‐stage worms were manually placed on fresh NGM plates (50 worms/plate, three replicates per condition). Worms were transferred daily to new plates, and survival was monitored by counting live and dead animals. Assays were conducted at 20°C. When testing different strains, N2 worms served as controls. Statistical significance was assessed using the log‐rank test (Schulz et al. 2007).

### 4.4 Motility Assay

The motility of the worms was measured on days 7, 14, and 21 using a wMicrotracker device (NemaMetrix, US) (Statzer et al. 2022). For each condition, at least three wells of a 96‐well plate were prepared, each containing 15 worms suspended in 90 μL of S‐buffer. Motility was recorded for 2 h at 20°C using the wMicrotracker software. The results were expressed as the percentage of body bends per minute over a 1 h measurement, normalized to control worms on day 7.

### 4.5 qRT‐PCR

Total RNA from *C. elegans* was extracted via S‐buffer washing, 3–4 freeze–thaw cycles (liquid nitrogen/37°C), and TRIzol (1 mL; Thermo Fisher) treatment. After adding 200 μL chloroform, samples were incubated (3 min) and centrifuged (120,000 rpm); the aqueous phase was collected, RNA precipitated with 500 μL isopropanol, centrifuged (10 min, 4°C), and the pellet resuspended in 25 μL RNase‐free water (Rio et al. 2010). RNA concentration was determined with a UviLine 9400 spectrophotometer (Schott). For cDNA synthesis, 1 μg RNA was reverse‐transcribed using the Applied Biosystems kit (Thermo Fisher) per the manufacturer's protocol. mRNA levels were measured via qRT‐PCR (QuantiFast SYBR Green; Qiagen). Act‐1 and HPRT1 (Qiagen, Cat No. QT0059066) were used as reference genes for nematode and human samples, respectively. Primers (see Table 2) were obtained from Invitrogen (Austria). qRT‐PCR was performed on a LightCycler 480 (Roche), and gene expression normalized to housekeeping genes and shown as relative expression.

**TABLE 2:** Primer sequences for qRT‐PCR.

Name	Forward	Reverse
*act‐1*	gctcttgccccatcaaccat	gccggactcgtcgtattctt
*mcu‐1*	cacaacaacagcctcctcaa	ggcaaggctcatttcttgac
*egl‐1*	caggacttctcctcgtgtgaagattc	gaagtcatcgcacattgctgcta
*ced‐3*	tgaaacagatgccagagatgaca	cgaaagagaactgggggaga
SOD1	ggcctgcatggattccatgttc	agtctccaacatgcctctcttc
SOD2	cctcacatcaacgcgcagatca	cctcggtgacgttcaggttgt
CAT	cctatcctgacactcaccgcca	agttggccactcgagcacggta

### 4.6 Quantification of H_2_O_2_

H_2_O_2_ levels were determined by the Amplex Red assay kit (Thermo Fisher Scientific, Austria), as described (Thomas et al. 2023). Synchronized and appropriately treated nematodes were incubated with 100 μM Amplex Red (Invitrogen, Carlsbad, USA) and 0.2 U/mL horseradish peroxidase in a sodium‐phosphate buffer for 3 h. Fluorescence was then measured using a CLARIOstar microplate reader (BMG Labtech, Germany). The results were normalized to protein content using the Pierce BCA protein assay (Thermo Fisher Scientific, Austria).

### 4.7 Basal Oxygen Consumption Rate (OCR)

Synchronized nematodes were diluted in M9 buffer, anesthetized with 25 μM tetramisole hydrochloride, and plated in Seahorse XF24 culture plates with approximately 40 worms per well before measuring basal OCR as described (Thomas et al. 2023).

### 4.8 Confocal Microscopy to Assess Mitochondrial Morphology and Glutathione Levels

Nematodes were anesthetized with 5 mM levamisole in S‐buffer on empty NGM plates, then immobilized on 0.05 μm Polybead‐coated slides under a 5% agar pad. Imaging was performed on a Nikon Eclipse Ti2 microscope with a 100×/1.45 NA oil objective, standard filters, and dual Kinetix CMOS cameras (PhotoMetrics, US). Mitochondrial imaging used the MIR151 strain (mitochondrial mCherry; HT1593 background). Excitation was at 435 nm (Celesta Light Engine; Lumencor, US); emission was collected at 501–521 nm. Images (2 × 2 binning, 500 ms exposure, 0.2 μm Z‐steps) were acquired at days 7, 14, and 21 of adulthood (10 worms/condition, 5 replicates). Images were blind deconvoluted, and mitochondrial morphology was analyzed with a custom ImageJ macro. Background subtraction (rolling ball) and global/local Otsu thresholding enabled segmentation. Subsequently, the 3D ImageJ Suite plugins (Ollion et al. 2013) were used to assess mitochondrial volume, surface area, compactness, and sphericity (Gottschalk et al. 2019).

To determine GSSG/GSH ratios, the JV2 nematode strain (jrIs2[Prpl‐17p::Grx1‐roGFP2+ unc‐119(+)]) expressing the Grx1‐roGFP2 biosensor under a ribosomal promoter was used. Measurements were performed using the Nikon Eclipse Ti2 microscope with the same optical setup. Excitation was provided by 400 nm and 475 nm laser light (Celesta, Light Engine; Lumencor, US), and emission was collected at 501–521 nm. Fluorescence signals were background‐subtracted using a defined region of interest (ROI), corrected for photobleaching via exponential decay fitting, and presented as F_475_/F_400_ (Tawfik et al. 2022).

### 4.9 Live‐Cell Imaging to Determine Mitochondrial Ca^2+^ and Cytosolic H_2_O_2_ Levels in Nematodes

To image the mitochondrial Ca^2+^ sensor YC3.60, the AQ3055 strain was analyzed using an inverted Olympus IX73 microscope (Olympus, Austria) equipped with a UPlanSApo 40×/1.25 silicon objective (Olympus, Austria), a CCD QImaging Retiga R1 camera (PhotoMetrics, US), and a LedHUB illumination system (Omicron‐Laserage Laserprodukte, Rodgau‐Dudenhof, Germany) with a 455/505 nm LED and a CFP/YFP filter set (Olympus, Austria). For FRET measurements, emission was split into two channels using a dual‐channel beam splitter DV2 (PhotoMetrics, US) and a CFP/YFP beamsplitter (505DCXR, Chroma Technologies, Bellows Falls, VT, USA). Basal mitochondrial Ca^2+^ levels were measured in S‐buffer for 2 min, followed by mitochondrial Ca^2+^ uptake assessment upon caffeine [15 mM] application. Data were background‐subtracted using a defined region of interest, corrected for photobleaching using an exponential decay fit, and presented as F_535_/_F480_.

Cytosolic H_2_O_2_ levels were measured in the JV1 strain (jrIs1 [rpl‐17p::HyPer + unc‐119(+)]) on day 14. Imaging was performed using the same Olympus IX73 microscope setup with a UPlanSApo 40×/1.25 silicon objective, CCD QImaging Retiga R1 camera, and a LedHUB illumination system equipped with a 455/505 nm LED and a CFP/YFP filter set. Excitation was achieved with either 455 nm or 505 nm, and emission was collected at 501–521 nm. Background subtraction was applied using a defined ROI, and bleaching correction was performed using an exponential decay fit. H_2_O_2_ measurement results are expressed as F_505_/F_455_ (Brugger et al. 2023).

### 4.10 Culturing, Seeding, and Treatment of Human Foreskin Fibroblasts HFF‐1

Human diploid fibroblasts (HFF‐1; ATCC SCRC‐1041) were cultured in DMEM (D5546; Sigma, Germany) with 10% heat‐inactivated FBS, 4 mM L‐glutamine, and 1% penicillin–streptomycin (Thermo Fisher, Austria) at 37°C, 5% CO_2_. Cells (passages 2–15) were passaged using 0.05% trypsin–EDTA, centrifuged (1500 rpm), counted in a Neubauer chamber, and seeded at the desired density (Cavinato et al. 2021). Mitoxantrone dihydrochloride (Merck, Germany) was prepared as a 10 mM DMSO stock. Cells were treated with 100 nM mitoxantrone [0.001% DMSO] or DMSO control for 3 h before measurements.

### 4.11 Cell Viability Analysis of HFF‐1 Cells

Cell viability was measured with the fluorometric CellTiter‐Blue Cell Viability Assay (Promega, Austria), which is based on the resazurin reduction method (Chen et al. 2018). HFF‐1 cells were seeded at a density of 3200 cells per well in flat‐bottom 96‐well plates. The cells were treated for 3 h with 100 nM mitoxantrone or DMSO as a control. Afterwards, the cells were washed two times with phosphate‐buffered saline (PBS), and 100 μL fresh media was added to each well. For the assay, 20 μL of CellTiter‐Blue reagent was added to four technical repeats of four different passages of either condition, 24 h or 48 h postmitoxantrone incubation. Following a 30‐min incubation at 37°C, fluorescence (excitation at 560 nm/emission at 590 nm) was measured, and cell viability was normalized to the respective control.

### 4.12 Proliferation Analysis of HFF‐1 Cells

Cell proliferation of fibroblasts was monitored using the CellCyte X—Live Cell Imager and Analyzer (Cytena GmbH, Germany) (Kalinova et al. 2025). HFF‐1 were seeded at a density of 40,000 cells per well in 12‐well plates. The cells were treated for 3 h with 100 nM mitoxantrone or DMSO as a control. Afterwards, the cells were washed twice with PBS, and 2 mL of fresh media was added to each well. Subsequently, the plates were placed into the CellCyte X—Live Cell Imager and Analyzer within an incubator (37°C, 5% CO_2_). Enhanced contour pictures of the fibroblasts were taken every 6 h, and the confluency was analyzed with the CellCyte Studio Software (Cytena GmbH, Germany).

### 4.13 Measurements of Mitochondrial Ca^2+^ and Mitochondrial H_2_O_2_ in HFF‐1 Cells

For live‐cell imaging, cells were seeded on 30 mm glass coverslips in 6‐well plates. Transfection with 0.5 μg mitoHyPer7 plasmid (Pak et al. 2020) and 3 μL Polyjet (SignaGen, US) in 1 mL serum−/antibiotic‐free medium was performed 2 days before imaging. To normalize maximal mitochondrial H_2_O_2_ levels, 150 μM H_2_O_2_ was added at the end of each measurement. Imaging was performed on a Nikon Eclipse Ti2 microscope (Nikon, Austria) with a 100×/1.45 NA oil objective, standard filters, and two Kinetix CMOS cameras (PhotoMetrics, US). Excitation was at 400 nm or 475 nm (Celesta Light Engine; Lumencor, US), and emission was collected at 501–521 nm. Data were background‐subtracted using a ROI and bleaching‐corrected via exponential decay fitting. H_2_O_2_ levels are shown as F_475_/F_400_ (Tawfik et al. 2022).

To assess mitochondrial Ca^2+^ levels, HFF‐1 were transfected via viral transduction with the FRET‐based mitochondrial Ca^2+^ sensor mtD1GO‐Cam (Waldeck‐Weiermair et al. 2012). After 2 days, the basal Ca^2+^ level and the response upon histamine stimulation were assessed on an inverted wide‐field microscope (Observer.A1; Carl Zeiss GmbH, Austria) as described previously (Waldeck‐Weiermair et al. 2012). Emission was collected with a 505dcxr beamsplitter on two sides of the camera (CCD camera, Coolsnap Dyno; PhotoMetrics, US). The biosensor mtD1Go‐Cam was excited with a wavelength of 477 nm (440AF21; Omega Optical, US), and emission was captured at 560 and 510 nm (480AF30 and 535AF26; Omega Optical, US). Results are shown as F_560_/_F510_.

### 4.14 Activity Assays of Antioxidant Defense Enzymes

HFF‐1 cells (100,000/well) were seeded in 6‐well plates and treated for 3 h with 100 nM mitoxantrone or DMSO (control). After two PBS washes, 2 mL fresh medium was added. At 24 h and 48 h posttreatment, cells were washed with ice‐cold PBS, scraped, and centrifuged (4 C, 2000 rpm, 8 min). Pellets were resuspended in 150 μL cold lysis buffer (50 mM potassium phosphate, 0.1 mM EDTA, 0.5% Triton X‐100), subjected to two freeze–thaw cycles, and centrifuged (12,000 rcf, 5 min, 4°C). Protein concentration was determined using the Pierce BCA Protein Assay Kit (Thermo Fisher). For SOD (MAK528) and catalase (219265) activity assays (Sigma‐Aldrich, Austria), 6 μg protein was used per reaction. Activities were measured according to the manufacturer's instructions, calculated via standard curves, and normalized to controls.

### 4.15 Mitochondrial Isolation

Nematodes were harvested from S‐buffer liquid culture on day 7, following treatment from day 4 with 25 μM 5‐fluorodeoxyuridine, 1 mM IPTG, and 100 μg/mL ampicillin (Roth, Austria), and the respective bacteria.

Worm pellets were resuspended in 5 mL IMBc buffer (200 mM sucrose, 10 mM Tris/MOPS, 10 mM EGTA/Tris, pH 7.4) and homogenized by passing them six times through an Isobiotec cell homogenizer (12 μm clearance; Isobiotec, Germany) using a 5 mL syringe. After centrifugation at 600 × g (15 min, 4°C) to remove debris, the supernatant was spun twice at 9000 × g (15 min, 4°C). The resulting mitochondrial pellet was resuspended in 100 μL IMBc for PDH assays or 300 μL MIR05 for respirometry (Sladowska et al. 2021).

HFF‐1 mitochondria were isolated in a similar manner. Cells were detached with 0.05% trypsin–EDTA, resuspended in 1 mL IMBc, and homogenized using the Isobiotec cell homogenizer with 6 μm clearance.

### 4.16 PDH Assay

A commercially available activity kit (ab109902; Abcam, US) was used to determine the PDH activity of isolated mitochondria from nematode samples. PDH was immunocaptured within the wells of a microplate, and the activity was determined by the reduction of NAD^+^ to NADH + H^+^, coupled to a reporter dye, which was measured at an absorbance of 450 nm. The protein concentration was determined by a BCA protein assay. Each sample was adjusted to 100 μg of protein per well. The assay is reflecting in a kinetic mode for 1 h. The rate (ΔmOD/min) was then calculated by using the following formula: Rate (mOD/min) = (Absorbance 2‐ Absorbance 1) / time (min) (Shimada et al. 2022).

### 4.17 Respirometry

Isolated mitochondria (50–100 μg) from nematodes and HFF‐1 cells were resuspended in 300 μL MIR05 respiration medium and added to an Oxygraph‐2 k respirometer chamber (Oroboros, Austria), prefilled with MIR05 at 20°C. Respiration was assessed using the SUIT‐008 protocol. Complex I activity was stimulated with 5 mM pyruvate, 2 mM malate, 2.5 mM ADP, and Mg^2+^. Membrane integrity was tested with 10 μM cytochrome c. Complex II was assessed after 0.05 μM rotenone inhibition using 10 mM glutamate and 10 mM succinate. Maximal respiration was induced with 0.5 μM CCCP. Complex IV activity was analyzed after inhibiting complexes I and III (0.5 μM rotenone, 2.5 μM antimycin A), using 2 mM ascorbate and 0.5 mM tetramethyl phenylenediamine (TMPD), followed by inhibition with 50 mM NaN_3_. O_2_ flux was normalized to protein content and analyzed using DatLab 7 (Oroboros) (Sladowska et al. 2021).

### 4.18 Statistical Analyses

Data were processed in Excel 2010 and analyzed using GraphPad Prism 9.3.1. Results are shown as mean ± SEM from at least three independent experiments. Two‐tailed Student's *t*‐tests were used for pairwise comparisons and one‐way ANOVA for multiple groups. For comparing significant distributions between different groups in the lifespan assays, statistical calculations were carried out using the log‐rank test. Nematode sample sizes are listed in Table 1. Cell sample sizes from live‐cell imaging are noted in the respective Figure's legend, providing the number of total cells measured and the number of independent experiments. Significance was set at *p ≤ 0.05, **p ≤ 0.01, ***p ≤ 0.001, and ****p ≤ 0.0001.

## Author Contributions

All authors participated in analyzing and interpreting the data. C.T.M.‐S. and D.B. designed the experiments. D.B., I.T., L.W., S.L., and F.M. performed lifespan assays. D.B., M.H., S.G., and K.K. performed live‐cell imaging experiments. J.O. performed qRT‐PCRs. K.Z. and M.R. established the novel worm strain MIR151. D.B. performed all other experiments. K.Z., M.R., E.M., M.H., and M.S. consolidated experimental procedures and edited the manuscript. C.T.M.‐S. supervised the project and wrote the manuscript in consultation with the other authors.

## Conflicts of Interest

The authors declare no conflicts of interest.

## Supporting information


**Figure S1:** acel70247‐sup‐0001‐FigureS1.tif.

**Figure S2:** acel70247‐sup‐0002‐FigureS2.tif.

**Figure S3:** acel70247‐sup‐0003‐FigureS3.tif.

**Figure S4:** acel70247‐sup‐0004‐FigureS4.tif.

**Figure S5:** acel70247‐sup‐0005‐FigureS5.tif.

## Data Availability

The data that support the findings of this study are available on request from the corresponding author. The data are not publicly available due to privacy or ethical restrictions.
