# Supplementary figures and images for: Enhancing Late‐Life Survival and Mobility via Mitohormesis by Reducing Mitochondrial Calcium Levels
Source: Aging Cell. 2025 Sep 26;24(11):e70247. doi: 10.1111/acel.70247 (PMC12608091; doi:10.1111/acel.70247)

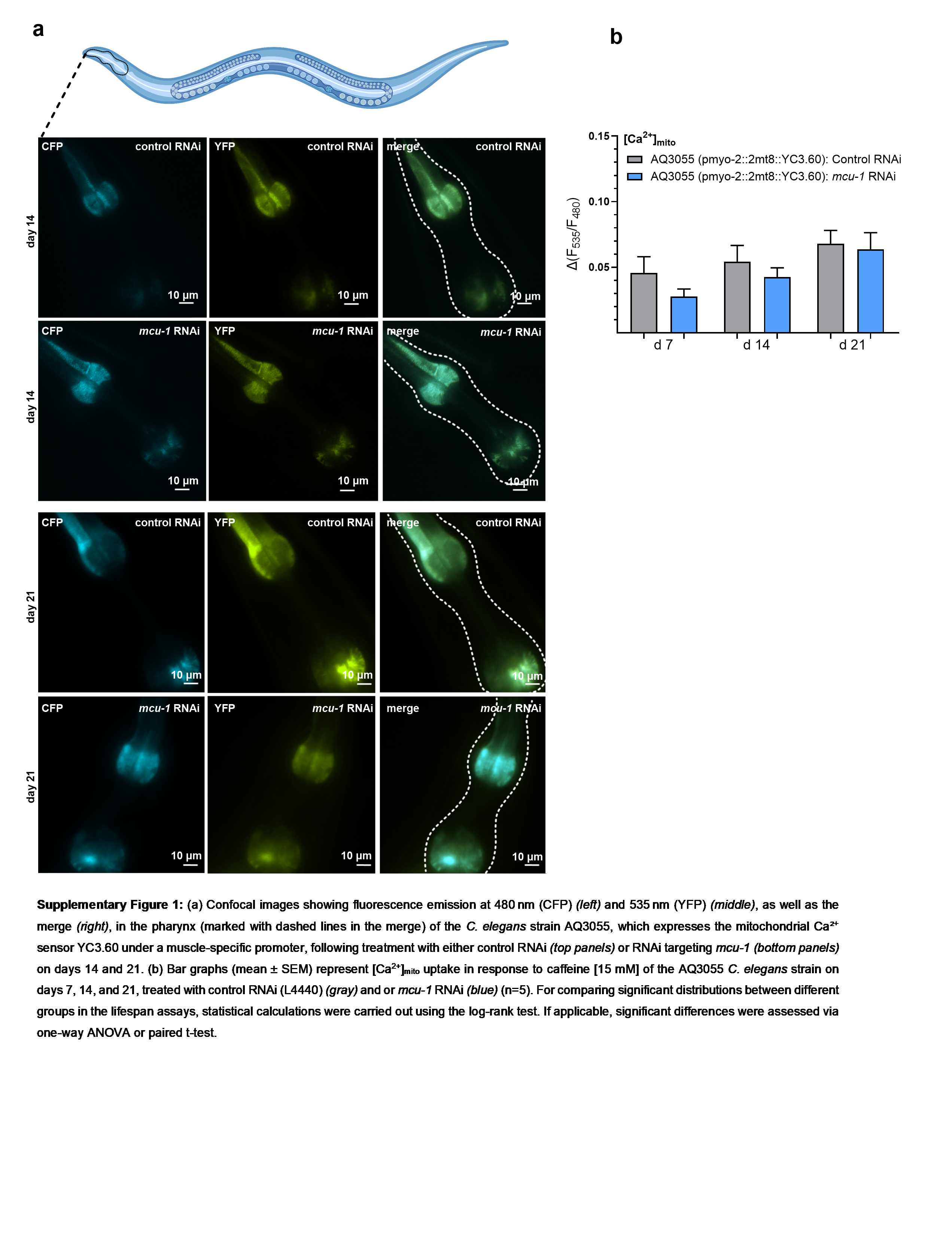

Supplement: Supplementary file 1 — Figure S1: acel70247‐sup‐0001‐FigureS1.tif. [file ACEL-24-e70247-s005.tif]

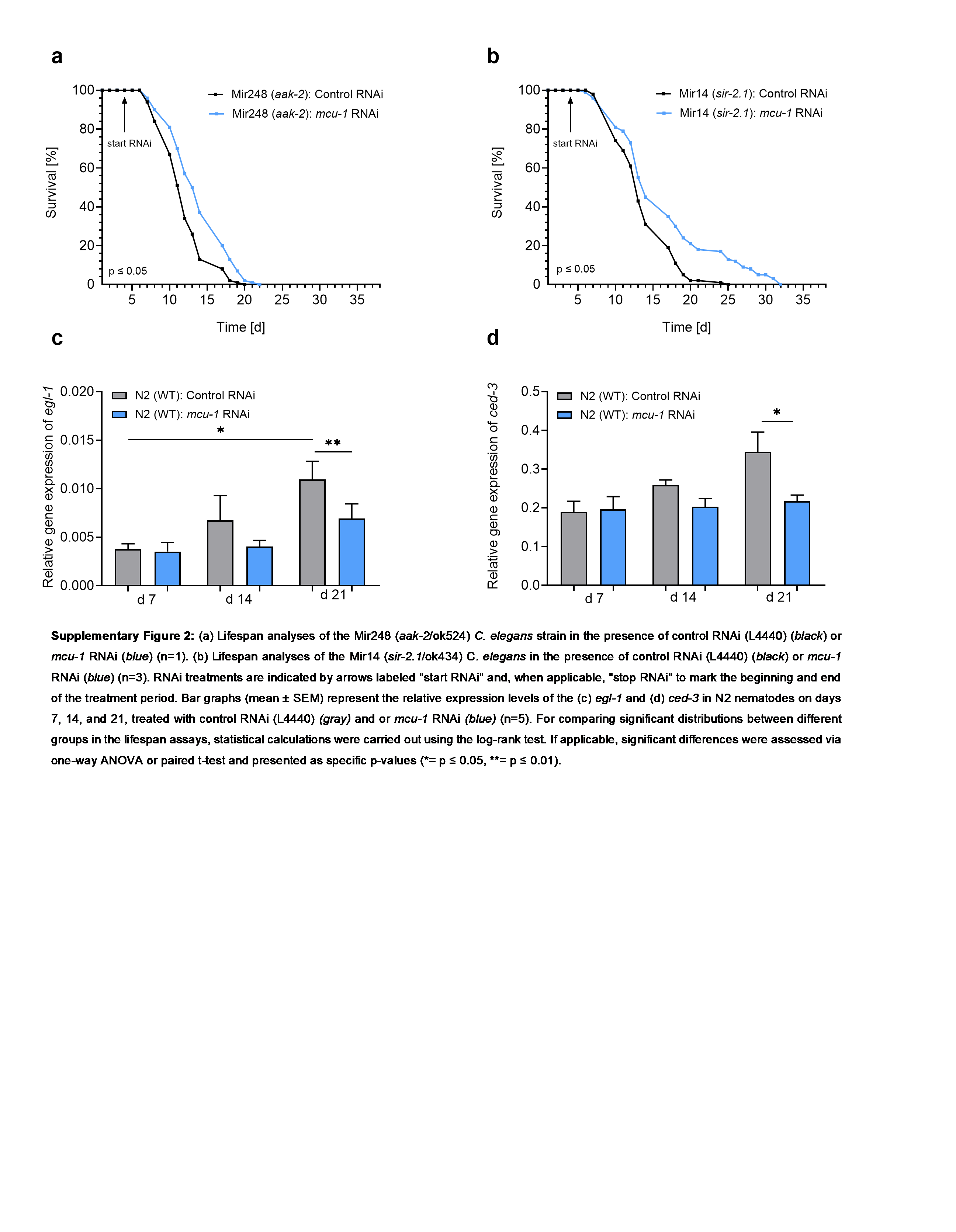

Supplement: Supplementary file 2 — Figure S2: acel70247‐sup‐0002‐FigureS2.tif. [file ACEL-24-e70247-s002.tif]

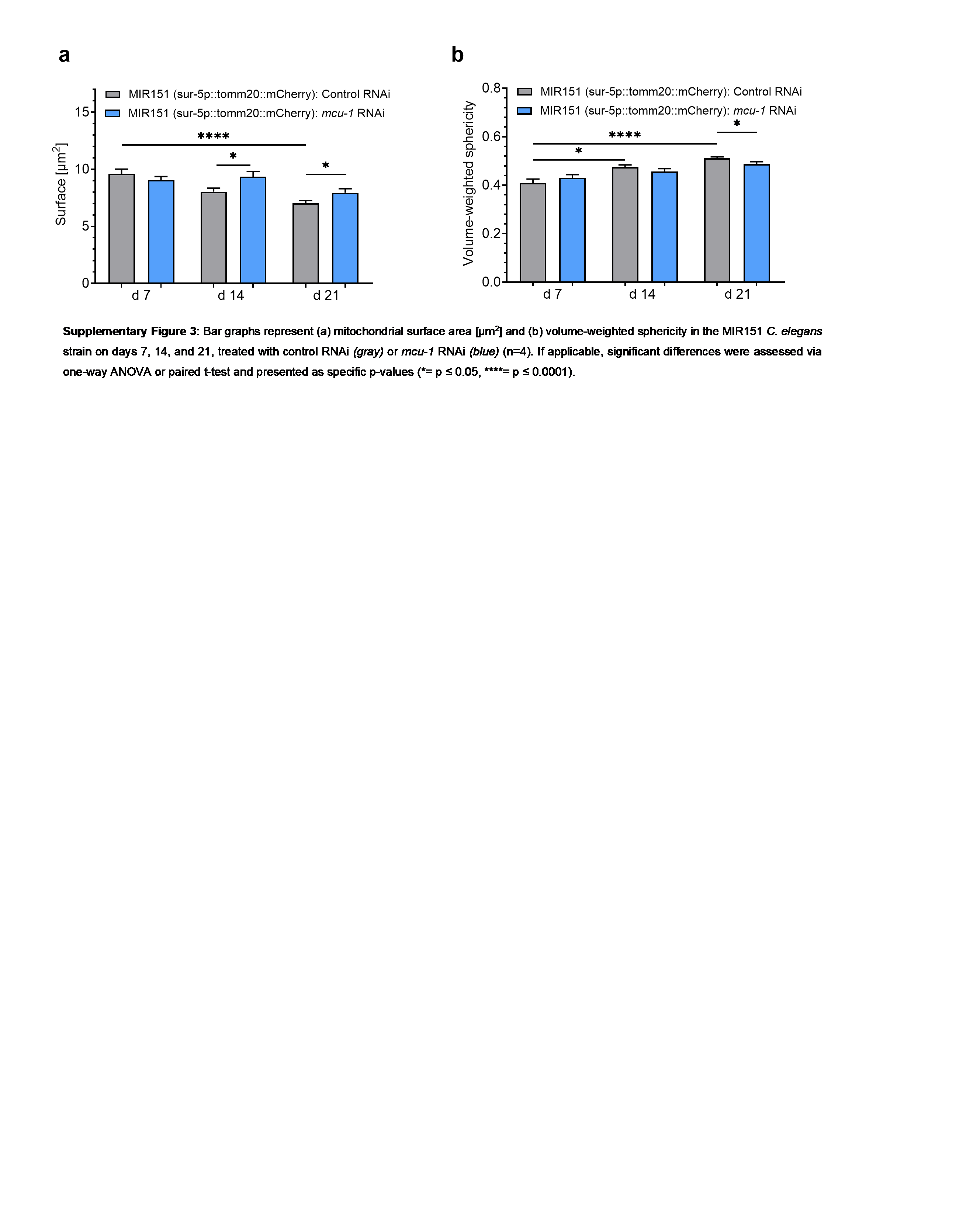

Supplement: Supplementary file 3 — Figure S3: acel70247‐sup‐0003‐FigureS3.tif. [file ACEL-24-e70247-s001.tif]

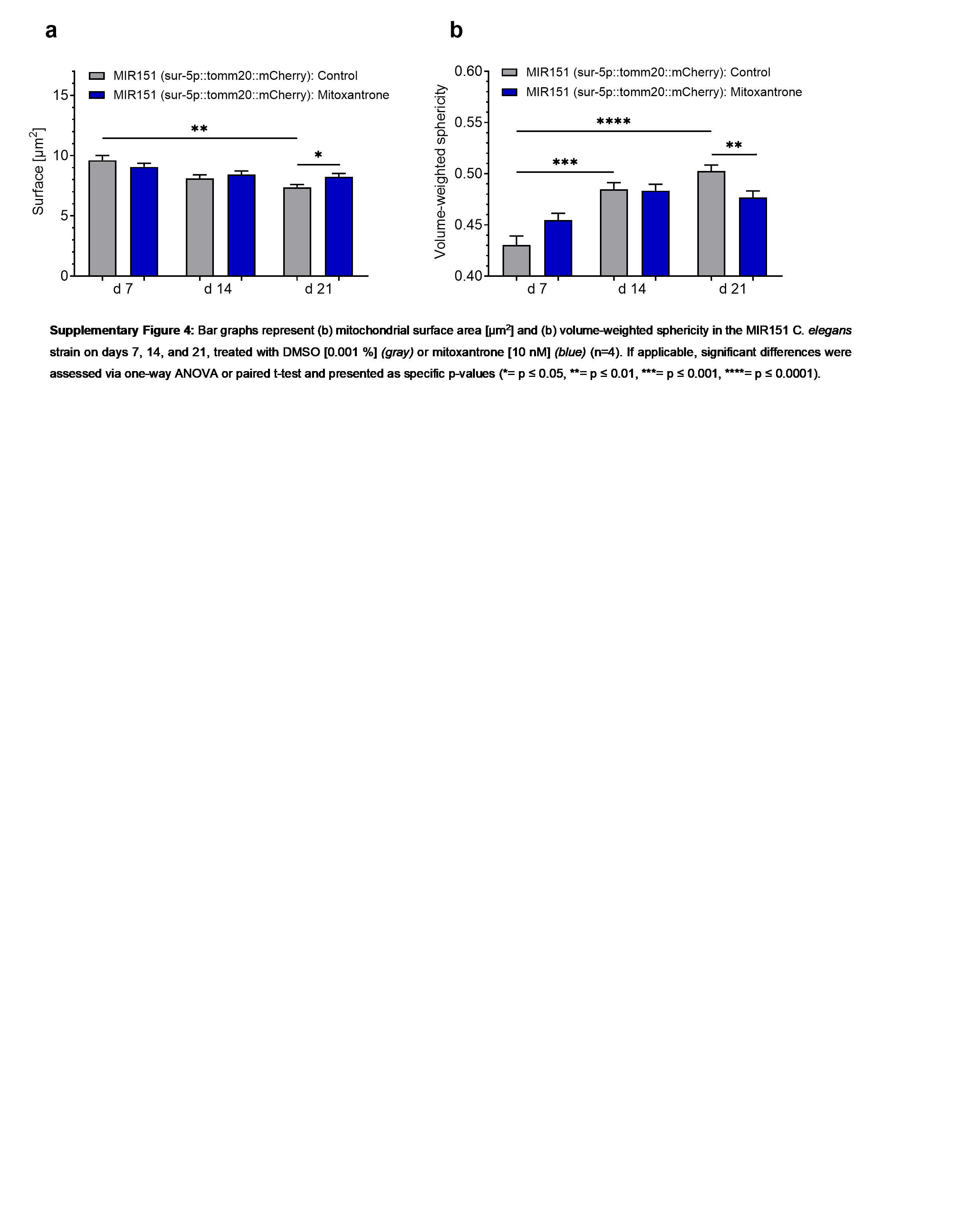

Supplement: Supplementary file 4 — Figure S4: acel70247‐sup‐0004‐FigureS4.tif. [file ACEL-24-e70247-s003.tif]

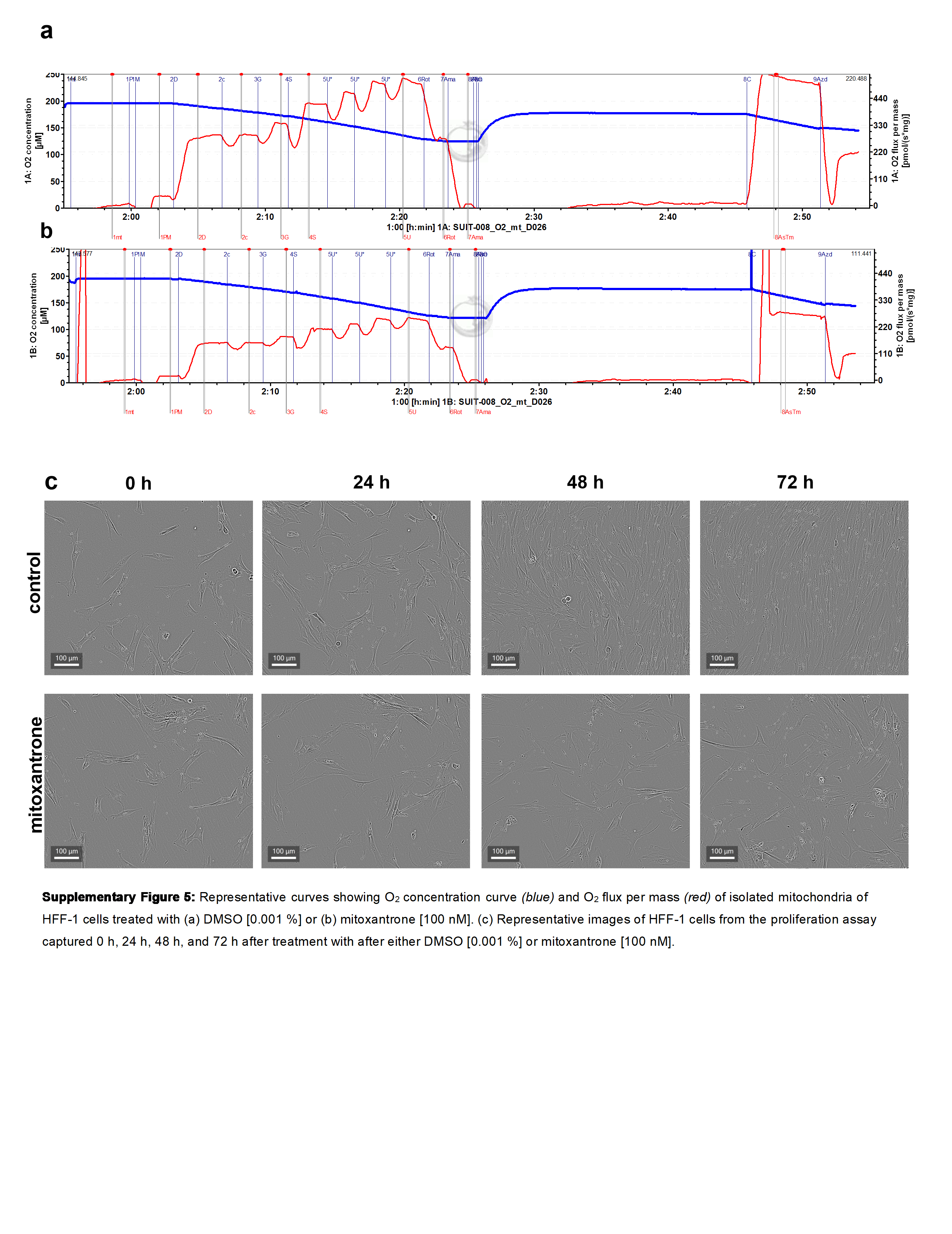

Supplement: Supplementary file 5 — Figure S5: acel70247‐sup‐0005‐FigureS5.tif. [file ACEL-24-e70247-s004.tif]
